# Quantum many-body mixed phase space revealed by hybrid feedback control

**DOI:** 10.1038/s41567-026-03431-z

**Published:** 2026-09-23

**Authors:** Hang Dong, Jie Ren, Andrew Hallam, Han Wang, Zhengyi Cui, Yiren Zou, Junlin Wang, Hekang Li, Qiujiang Guo, Zhen Wang, Lei Ying, Zlatko Papić

**Affiliations:** 1 School of Physics, ZJU-Hangzhou Global Scientific and Technological Innovation Center, and Zhejiang Key Laboratory of Micro-nano Quantum Chips and Quantum Control, Zhejiang University, Hangzhou, China; 2 School of Physics and Astronomy, University of Leeds, Leeds, UK

**Keywords:** Quantum simulation, Nonlinear phenomena

## Abstract

Understanding how complex systems transition between order and chaos is a central challenge of non-equilibrium physics. Although weak perturbations of classical integrable systems give rise to a mixed phase space in which regular and chaotic trajectories coexist, analogous behaviour in interacting quantum many-body systems has been difficult to identify. Here we develop and experimentally implement a hybrid quantum–classical feedback protocol that autonomously discovers and stabilizes long-lived regular trajectories in a superconducting quantum processor. Each iteration combines short-time quantum evolution with classical optimization that projects the dynamics back onto a low-entanglement variational manifold, effectively distilling coherence from chaotic evolution. The stabilized trajectories reveal signatures of a quantum many-body mixed phase space emerging from nonlinear variational dynamics, without a direct analogue in few-body quantum systems described by a small number of effective degrees of freedom. Our results establish a framework for the algorithmic identification and control of coherent dynamics in quantum many-body systems.

## Abstract (web summary)

Regular and chaotic trajectories can coexist in classical systems, but identifying an equivalent in quantum many-body dynamics is difficult. A feedback protocol on a superconducting processor now reveals and stabilizes such mixed dynamics.

## Main

The coexistence of regular and chaotic motion is a universal feature of dynamical systems, ranging from planetary orbits and ocean waves to turbulence and transport in complex materials^1,2^. In classical mechanics, this interplay is governed by the Kolmogorov–Arnold–Moser (KAM) theorem^3^, which shows that weak perturbations of integrable systems preserve a finite measure of invariant tori, producing a mixed phase space in which regular and chaotic trajectories coexist. In the quantum realm, the remnants of integrability can survive in few-body systems described by a single collective degree of freedom^4^ or under weak perturbations of models solvable by the Bethe ansatz^5^. However, strongly interacting quantum systems can exhibit qualitatively new forms of dynamics that resemble the mixed phase space but have no direct few-body analogue. In such systems, the coexistence of coherent and chaotic motion may arise from the collective evolution of entangled degrees of freedom rather than from a conventional classical limit (*ℏ* → 0) (ref. ^6^).

The process of thermalization in generic quantum systems is governed by the eigenstate thermalization hypothesis (ETH)^7,8^, which states that highly excited eigenstates reproduce the thermal expectation values of local observables. Systems that weakly break the ETH, for example, those with quantum many-body scars and Hilbert-space fragmentation^9–11^, are a natural starting point to search for quantum many-body analogues of the mixed phase space as these systems are generally chaotic yet host special non-thermalizing states^12,13^. However, such initial states are exceedingly rare in the many-body Hilbert space and, thus, difficult to identify. A framework capable of revealing and stabilizing non-thermal trajectories is, therefore, needed to access new universality classes of non-ergodic quantum dynamics beyond currently known ETH violations.

Here we demonstrate a hybrid quantum–classical feedback protocol that autonomously discovers and stabilizes coherent trajectories in interacting many-body systems. Each iteration of the protocol alternates between short-time quantum evolution and classical optimization that steers the system back towards a low-entanglement variational manifold^14^. The evolution generically drives the state outside the manifold, generating entanglement and exploring directions in Hilbert space that are not captured by the variational ansatz; consequently, the projection step depends on genuinely quantum many-body dynamics rather than on evolution restricted to the manifold alone. Repeated application of this evolution–projection cycle filters out the entangling behaviour of chaotic motion, converging to a stable periodic orbit if it exists. Conceptually, the method blends the time-dependent variational principle (TDVP)^15–18^ with active feedback control^19,20^ by transforming the geometric projection of quantum dynamics into a practical measurement-based algorithm, suitable for current quantum hardware. In particular, we can perform quantum evolution in the analogue mode, which substantially suppresses errors associated with Trotter gate decompositions.

We implement the hybrid feedback scheme on a programmable superconducting qubit processor that realizes an interacting Su–Schrieffer–Heeger (SSH) ladder^21,22^. By mapping the ladder’s dynamics onto a shallow variational circuit and monitoring the local fidelity, we uncover islands of regular motion within a chaotic sea utilizing a many-body analogue of the Poincaré section. The resulting trajectories provide the fingerprints of quantum many-body mixed phase space, which was previously proposed to occur in Rydberg atom arrays^23^ but has so far eluded experimental observation. This emergent structure originates from nonlinear TDVP dynamics and many-body correlations rather than the conventional classical limit, serving as an emergent semiclassical phase-space picture that bridges classical chaos and quantum ergodicity breaking.

## Superconducting circuit with mixed phase space

**Fig. 1:**
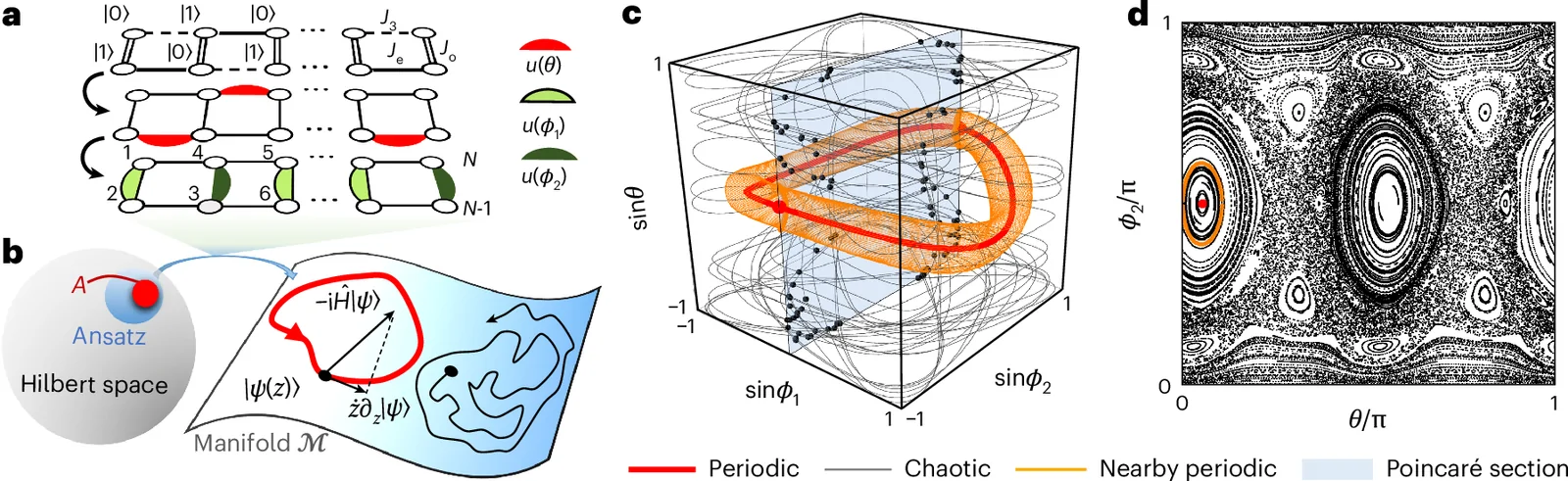
Superconducting circuit with a mixed phase space. **a**, Shallow variational circuit used to approximate the real-time dynamics of an interacting SSH ladder (equation (1)) on our superconducting qubit processor. The inter-leg coupling *J*_o_ is uniform, whereas the intra-leg couplings alternate between *J*_e_ and *J*_3_, defining an SSH-type dimerization pattern. The calibrated device couplings are *J*_e_/2π = 3.0 MHz, *J*_3_/2π = 0.25 MHz and *J*_o_/2π = 5.0 MHz. Starting from the Néel-ordered product state, two layers of entangling XY gates are applied: first $\hat{u}(\theta )$ on even bonds, followed by alternating $\hat{u}({\phi }_{1})$ and $\hat{u}({\phi }_{2})$ on odd bonds (see the main text). **b**, Circuit ansatz from **a** (equation (2)) defines a three-dimensional variational manifold ${\mathcal{M}}$ (blue region) embedded in the full Hilbert space, parameterized by **z** = (*θ*, *ϕ*_1_, *ϕ*_2_), which hosts a low-dimensional projection of quantum dynamics. The exact Schrödinger evolution, $-i\hat{H}\left.| \psi \right\rangle$, is projected onto the tangent space of ${\mathcal{M}}$ using TDVP. The red region labelled *A* illustrates a subset of the ansatz states associated with the stable periodic-orbit island of the TDVP flow. Depending on the initial conditions, the projected trajectory may form a regular orbit (red curve) or wander chaotically through the manifold (black curve). **c**, Representative classical trajectories in the reduced parameter space (*θ*, *ϕ*_1_, *ϕ*_2_), obtained by integrating the TDVP equations of motion (see Methods for the explicit expressions). A stable periodic orbit (red), a nearby regular trajectory (orange) and a chaotic trajectory (grey) are shown. The plane ${\phi }_{1}=0\,\mathrm{mod}\,\,2\uppi$ (light blue) marks the slice defining the Poincaré section. **d**, Poincaré section obtained from the intersections of TDVP trajectories with the plane ${\phi }_{1}=0\,\mathrm{mod}\,\,2\uppi$ in **c**. The section is generated from 350 trajectories evolved up to a late time *t* = 2,000 (in units *ℏ*∣*J*_o_∣ = 1), each initialized from random points on the manifold. Regular trajectories form discrete point sets lying along smooth invariant curves (the cross-sections of tori); two sets of representative points are highlighted in red and orange. Black points include both chaotic trajectories, which populate extended regions, and unhighlighted regular points on other tori. The section reveals the coexistence of regular islands and a chaotic sea within the TDVP dynamics. Source data

The versatility of superconducting qubit platforms, including high-fidelity initialization, tunable two-qubit couplings and local measurements, make it possible to access both ergodic and non-ergodic dynamical regimes of quantum many-body systems and resolving physical observables at the single-qubit level. In this work, we exploit these capabilities to design an interacting spin-1/2 model inspired by the SSH chain^21^ that hosts a many-body analogue of the mixed phase space. Our model is realized on a square ladder with *N* = 24 qubits, schematically illustrated in Fig. 1a. The circuit encodes the following Hamiltonian:

1 $$\begin{array}{lll}\hat{H}&=&-\mathop{\sum }\limits_{n=1}^{N/2-1}\left({J}_{n}^{\mathrm{t}}\,{{\mathrm{XY}}}_{(n,1),(n+1,1)}+{J}_{n}^{\mathrm{b}}\,{{\mathrm{XY}}}_{(n,2),(n+1,2)}\right)\\ &&-\mathop{\sum }\limits_{n=1}^{N/2}{J}_{n}^{{\mathrm{int}}}\,{{\mathrm{XY}}}_{(n,1),(n,2)}\,,\end{array}$$

where (*n*, *r*) labels the *n*th qubit in the row *r* ∈ {1, 2} and ${{\rm{XY}}}_{i,\,j}\equiv {\hat{\sigma }}_{i}^{+}{\hat{\sigma }}_{j}^{-}+{\hat{\sigma }}_{j}^{+}{\hat{\sigma }}_{i}^{-}$ denotes an exchange interaction in terms of the standard Pauli raising and lowering operators, ${\hat{\sigma }}^{\pm }$. The inter-leg couplings are chosen uniformly, ${J}_{n}^{{\mathrm{int}}}={J}_{\mathrm{o}}$, whereas intra-leg couplings alternate as ${J}_{2n}^{\mathrm{t}}={J}_{2n+1}^{\mathrm{b}}={J}_{\mathrm{e}}$ and ${J}_{2n+1}^{\mathrm{t}}={J}_{2n}^{\mathrm{b}}={J}_{3}$. For convenience, we map the ladder circuit onto a one-dimensional chain that can be conveniently labelled by a single-site index (Fig. 1a)—a notation we will adopt throughout this paper.

Different choices of the couplings allow us to smoothly interpolate between integrable and chaotic regimes. For instance, when *J*_e_ = *J*_3_ = 0, the circuit breaks up into decoupled dimers, which support exactly periodic rung oscillations, such as from a product state $\left\vert \varphi \right\rangle =\left\vert 01100110\ldots \,\right\rangle$. When *J*_e_ and *J*_3_ are non-zero, the system is chaotic, yet the same initial state continues to approximately oscillate, as previously observed in ref. ^22^. In Supplementary Section 9 and Supplementary Fig. 13a, we demonstrate that the dynamics of the model in equation (1) are much richer: even in the thermodynamic limit (*N* → ∞), the model displays a breakdown of ETH: it hosts continuous families of initial conditions at the same energy density, yet with starkly different slopes of entanglement growth. Below, we develop a framework that allows us to interpret this breakdown as a many-body manifestation of the mixed phase space.

To capture the coexistence of coherent and thermalizing dynamics, we introduce a shallow variational circuit ansatz that serves as an effective low-dimensional representation of the full quantum dynamics. The ansatz consists of two-qubit entangling gates ${\hat{u}}_{i,\,j}(\theta )=\exp (i\theta \,{{\rm{XY}}}_{i,\,j})$ applied sequentially along the lattice, starting from a Néel-ordered product state. We first apply $\hat{u}(\theta )$ on all even bonds to form the first layer operator ${\hat{U}}_{1}(\theta )={\hat{u}}_{2,3}(\theta ){\hat{u}}_{4,5}(\theta )\cdots \,$, followed by alternating $\hat{u}({\phi }_{1})$ and $\hat{u}({\phi }_{2})$ on odd bonds, which define the second layer operator ${\hat{U}}_{2}({\phi }_{1},{\phi }_{2})={\hat{u}}_{1,2}({\phi }_{1}){\hat{u}}_{3,4}({\phi }_{2})\ldots \,$ (Fig. 1a). The resulting shallow circuit ansatz is

2 $$\left.| \psi ({\bf{z}})\right\rangle \equiv {\hat{U}}_{{\mathcal{M}}}({\bf{z}})\left.| 0101\ldots \,\right\rangle ,\quad {\hat{U}}_{{\mathcal{M}}}({\bf{z}})={\hat{U}}_{2}({\phi }_{1},{\phi }_{2}){\hat{U}}_{1}(\theta ),$$

where we introduced the collective label for the circuit parameters **z** ≡ (*θ*, *ϕ*_1_, *ϕ*_2_).

The ansatz in equation (2) is motivated by the first-order Trotterization of the SSH ladder Hamiltonian without the *J*_3_ term, making it quantitatively accurate in the regime *J*_e_, *J*_3_ ≪ *J*_o_ and for short evolution times. It provides a faithful description of the low-entanglement dynamics responsible for coherent oscillations and remains sufficiently expressive to capture the coexistence of regular and chaotic trajectories. The choice of the ansatz is an important ingredient of the construction. Different variational manifolds provide different approximations to the full quantum dynamics and may highlight different aspects of the resulting phase-space structure. The shallow circuit considered here is motivated by both the underlying SSH Hamiltonian and experimentally accessible operations.

The set of all states generated by the circuit in equation (2) spans a three-dimensional manifold ${\mathcal{M}}=\{\,\left.| \psi ({\bf{z}})\right\rangle \,| \,{\bf{z}}\in {\left[0,2\uppi \right)}^{3}\}$ embedded within the exponentially large Hilbert space (Fig. 1b). We use TDVP^15^ to describe the time evolution within ${\mathcal{M}}$: the exact Schrödinger dynamics are projected onto the tangent space of ${\mathcal{M}}$, yielding effective classical equations of motion for the variational parameters **z**. This phase-space representation is constructed in terms of variational parameters within the manifold rather than canonical coordinates; hence, it should be viewed as an emergent description that organizes the underlying many-body dynamics. The equations of motion are nonlinear, reflecting the restricted structure of the manifold and its geometry (Methods provides their explicit form and Supplementary Section 4 provides the full derivation).

Within the TDVP framework, the projected dynamics in the **z** space reveal qualitatively different behaviours depending on the initial state. As illustrated in Fig. 1c, some trajectories remain confined to quasi-periodic orbits, reminiscent of KAM tori, whereas others wander irregularly and spread throughout the parameter space. The coexistence of these regular and irregular trajectories is a defining feature of the mixed phase space. A powerful visualization tool of this coexistence is the Poincaré section^1^: by recording points each time a trajectory intersects a chosen plane, for example, ${\phi }_{1}=0\,\mathrm{mod}\,\,2\uppi$, the effective dynamics are reduced to a two-dimensional slice. As shown in Fig. 1d, regular trajectories appear as smooth closed curves corresponding to slices of invariant tori, since the orbit continues to intersect the plane in the vicinity of the original location even at late times. By contrast, chaotic trajectories manifest as scattered points filling extended regions, since chaotic dynamics take the system far away from the original intersection at later times. As we will show below, the location, size and fine structure of these features depend sensitively on (*J*_o_, *J*_e_, *J*_3_), in analogy with the KAM theorem in classical mechanics.

The shallow circuit ansatz combined with TDVP projection provides a compact description of the interacting quantum model (equation (1)), displaying hallmarks of classical nonlinear systems, such as periodic trajectories embedded in a chaotic background. One may wonder whether the same features could be captured within the conventional semiclassical description based on large-*S* spin coherent states. This is not possible because the entanglement between neighbouring spins is essential for describing their dynamics. On the other hand, the variational manifold approach naturally represents a type of semiclassical limit: weakly entangled ansatz form an overcomplete basis of the Hilbert space, and the TDVP equations correspond to the saddle-point trajectories of a path integral over these states^24^. In this sense, the trajectories generated by the circuit shown in Fig. 1 are in direct correspondence with full quantum dynamics, as we demonstrate next.

## Mapping the Poincaré section

Although the TDVP framework allows us to visualize the mixed phase space through Poincaré sections, such diagnostics are not directly accessible in experiment: constructing a Poincaré section requires tracking the effective phase-space variables **z**, which are emergent coordinates of the variational manifold rather than physical observables of the qubits. Signatures of regular and chaotic behaviour can, in principle, be revealed in the dynamics of local observables. However, due to the limited coherence time of the hardware, such quantities may not be sufficiently sensitive to reveal well-defined KAM tori (Fig. 1d). To overcome these limitations, we introduce a closely related and experimentally accessible quantity based on imbalance of a subsystem *A*:

3 $${{\mathcal{I}}}_{{\bf{z}}}(t)=\frac{1}{N_{A}}\sum _{j\in A}{(-1)}^{\,j-1}\langle \psi (t)| {\hat{U}}_{{\mathcal{M}}}({\bf{z}}){\hat{\sigma }}_{j}^{z}{\hat{U}}_{{\mathcal{M}}}^{\dagger }({\bf{z}})| \psi (t)\rangle .$$

**Fig. 2:**
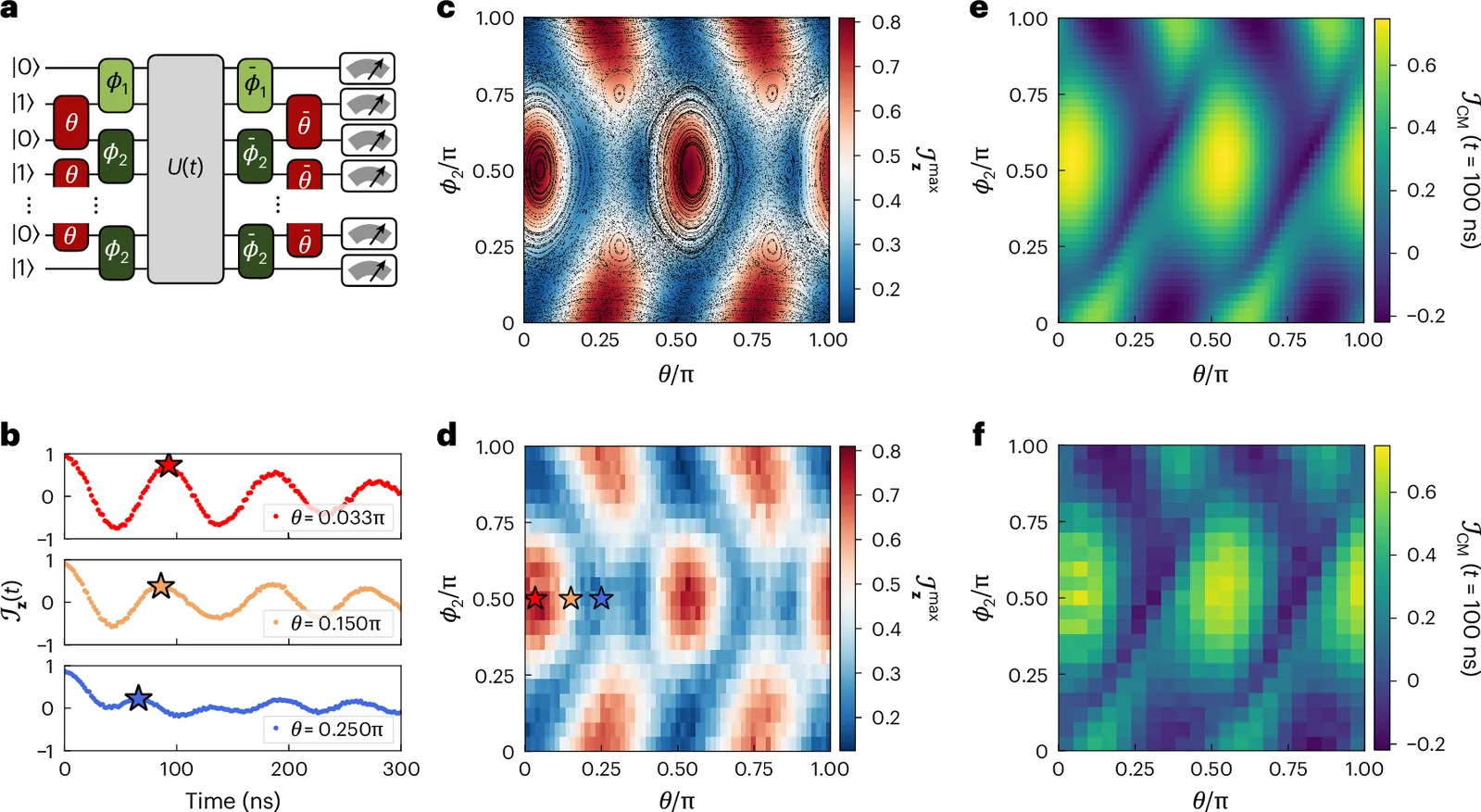
Experimental mapping of the Poincaré section. **a**, Quantum circuit implementing the measurement of imbalance ${{\mathcal{I}}}_{{\bf{z}}}(t)$ in equation (3). The system is initialized in a product state parameterized by (*θ*, *ϕ*_1_, *ϕ*_2_) by acting with a unitary ${\hat{U}}_{{\mathcal{M}}}$ in equation (2), then evolved by the unitary $\hat{U(t)}$ generated by the Hamiltonian in equation (1) and finally acted on by the inverse circuit ${\hat{U}}_{{\mathcal{M}}}^{\dagger }$ before read-out. **b**, Imbalance dynamics ${{\mathcal{I}}}_{{\bf{z}}}(t)$ for three representative initial states with fixed *ϕ*_2_ = 0.5π and *θ*/π = 0.033, 0.150 and 0.250 (red, orange and blue, respectively). The star on each curve indicates the time at which the first revival peak is identified; this defines the quantity ${{\mathcal{I}}}_{{\bf{z}}}^{\max }$ whose values are shown across the phase space in **c** and **d**. **c**,**d**, First revival peak of the subsystem imbalance, ${{\mathcal{I}}}_{{\bf{z}}}^{\max }$, mapped across the phase space (*θ*, *ϕ*_2_) with *ϕ*_1_ = 0. **c**, Numerical results obtained from the exact diagonalization of a 16-qubit chain with periodic boundary conditions, overlaid with the TDVP Poincaré section (black dots), illustrating the close correspondence between the two. **d**, Corresponding experimental measurement on the quantum processor using 24 qubits. The three initial conditions in **b** are marked in **d** by red, orange and blue stars, indicating their locations within the regular island and the surrounding chaotic region. The system is evolved for a sufficient amount of time (between 50 and 120 ns) to capture the first peak of ${{\mathcal{I}}}_{{\bf{z}}}(t)$. **e**,**f**, Co-moving imbalance ${{\mathcal{I}}}_{{\mathrm{CM}}}$, representing the imbalance of the full quantum state along the classical trajectory predicted by TDVP, evaluated at a fixed time *t* = 100 ns. **e**, Numerical result. **f**, Data from the quantum processor. The regular trajectories remain confined to the manifold, whereas chaotic trajectories experience pronounced leakage outside the manifold. In **b**–**f**, the imbalance was calculated for the 12 qubits in the middle of the chain, with each experimental data point averaged over 300 independent shots. Source data

The corresponding measurement protocol is summarized in Fig. 2a: the variational states $\left\vert \psi ({\bf{z}})\right\rangle$ are prepared with the help of the unitary ${\hat{U}}_{{\mathcal{M}}}$ in equation (2), evolved under the Hamiltonian (equation (1)) using the calibrated couplings and scheme shown in Fig. 1, and subsequently reversed by an inverse unitary ${\hat{U}}_{{\mathcal{M}}}^{\dagger }$ before measuring the imbalance. We note that the evolution *U*(*t*) is implemented in an analogue (non-Trotterized) manner using continuously activated couplings, thereby avoiding errors associated with discrete gate decompositions, whereas the circuit representation shown in Fig. 2a serves only to illustrate the logical structure of the protocol.

**Extended Data Fig. 1:**
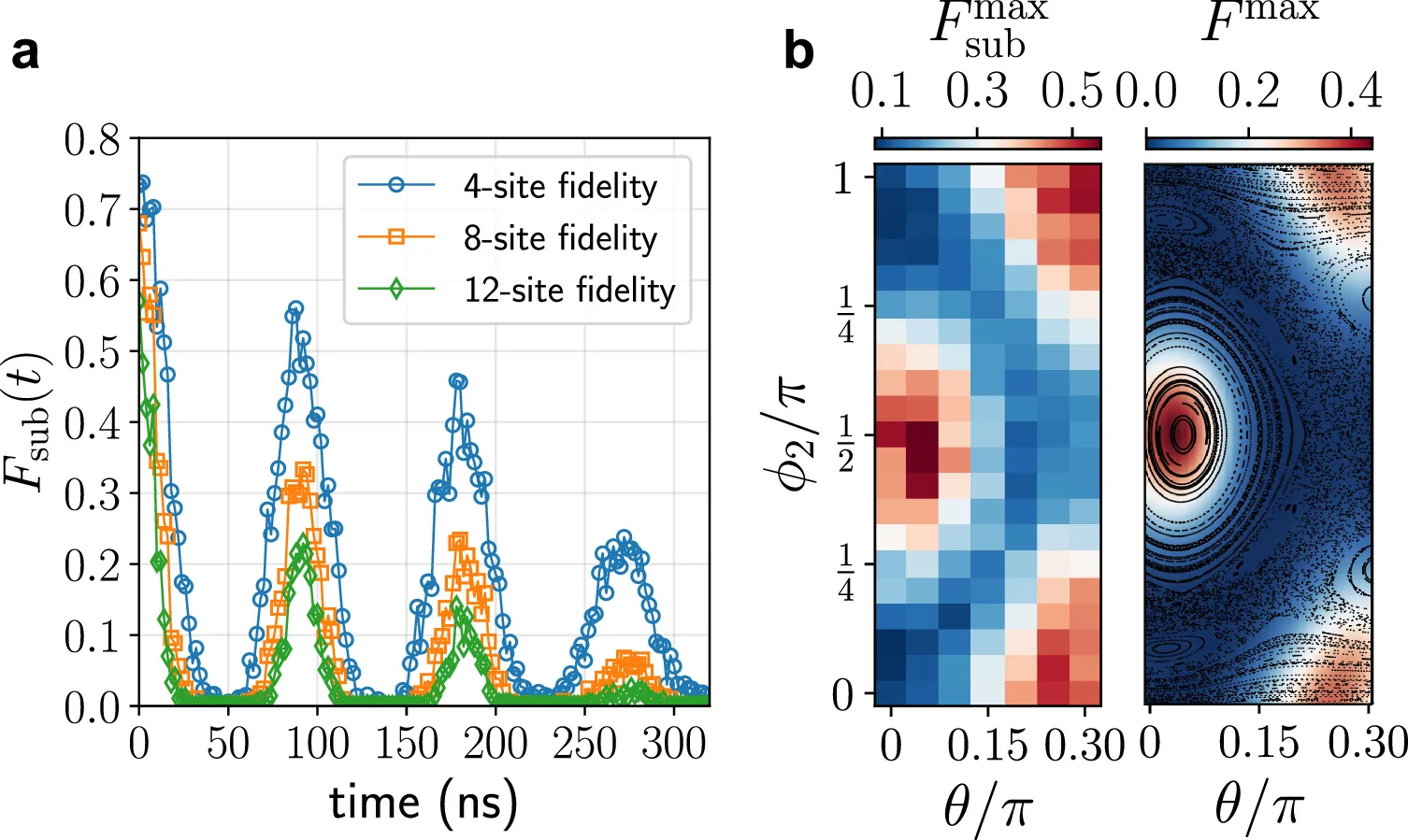
Subsystem fidelity. **a**, Subsystem fidelity F_sub_(t) for three choices of subsystem size L_sub_ = 4, 8, 12 and fixed initial condition (*θ*, *ϕ*_2_) = (0.05*π*, 0.5*π*). In all the measurements, the subsystem is taken as the central block of L_sub_ qubits. **b**, Poincare-section-like maps constructed from the first fidelity revival. The left panel shows the 4-qubit subsystem fidelity ${{\rm{F}}}_{{\rm{sub}}}^{\max }(\theta ,\,{\phi }_{2})$ obtained experimentally. The right panel shows the corresponding global fidelity ${{\rm{F}}}^{\max }(\theta ,\,{\phi }_{2})$ obtained from exact diagonalization of an L = 16 spin chain with periodic boundary conditions, overlaid with the TDVP Poincare section (black dots). Despite the larger fluctuations in the fidelity-based maps, the underlying phase-space structure, including the regular islands and chaotic regions, is visible in both cases. Larger subsystems more closely approximate the global fidelity but also exhibit increased noise. The experimental bitstring statistics were acquired using 1200 shots per point. Source data

To gain some intuition about ${{\mathcal{I}}}_{{\bf{z}}}$, note that for the trivial circuit ${\hat{U}}_{{\mathcal{M}}}({\bf{0}})=\hat{{\mathbb{1}}}$, ${{\mathcal{I}}}_{{\bf{0}}}$ simply measures the difference between the occupations of odd and even sites, that is, staggered magnetization. For general parameters **z**, ${{\mathcal{I}}}_{{\bf{z}}}$ quantifies the overlap between the quantum state $\left.| \psi \right\rangle$ and the variational ansatz $\left.| \psi ({\bf{z}})\right\rangle$. If $\left.| \psi \right\rangle$ is given by equation (2), applying the inverse circuit ${\hat{U}}_{{\mathcal{M}}}^{\dagger }({\bf{z}})$ maps it back to the Néel state, maximizing the imbalance. Consequently, during the dynamics, ${{\mathcal{I}}}_{{\bf{z}}}(t)=1$ if and only if ∣〈*ψ*(0)∣*ψ*(*t*)〉∣^2^ = 1. Thus, ${{\mathcal{I}}}_{{\bf{z}}}$ serves as a practical proxy for global state fidelity, with the advantage of smaller measurement fluctuations compared with the latter (Methods and Extended Data Fig. 1). Furthermore, ${{\mathcal{I}}}_{{\bf{z}}}$ is conveniently normalized: starting from an ansatz state $\left.| \psi ({\bf{z}})\right\rangle$, ${{\mathcal{I}}}_{{\bf{z}}}(t=0)$ is initially equal to unity and takes values strictly within the interval [−1, 1] at later times, providing a consistent scale for comparing different initial conditions. This normalization, together with its direct experimental accessibility, makes the imbalance an ideal observable for probing the underlying mixed structure of the dynamics.

In Fig. 2b–f, each initial ansatz state is evolved under the Hamiltonian in equation (1) without variational projection. Figure 2b compares the imbalance dynamics ${{\mathcal{I}}}_{{\bf{z}}}(t)$ for three representative initial states, with the subsystem *A* being the central 12 qubits. When the system is initialized within a regular region of the TDVP phase space, ${{\mathcal{I}}}_{{\bf{z}}}(t)$ displays long-lived coherent oscillations with pronounced revivals characteristic of quasi-periodic motion. By contrast, an initial state chosen from the chaotic region exhibits a much faster decay of ${{\mathcal{I}}}_{{\bf{z}}}(t)$ with strongly suppressed revivals, signalling fast thermalization. We define the first revival peak ${{\mathcal{I}}}_{{\bf{z}}}^{\max }$ as the value of ${{\mathcal{I}}}_{{\bf{z}}}(t)$ at its first local maximum, and use it as a measure of dynamical regularity for each initial condition. Scanning over (*θ*, *ϕ*_2_) yields a Poincaré-section-like map constructed entirely from local observables, that is, without reconstructing the full variational coordinates (*θ*, *ϕ*_1_, *ϕ*_2_). Figure 2c,d presents a direct comparison between numerical simulations and experimental measurements of the Poincaré section. In both cases, trajectories initialized within regular regions of the TDVP phase space (Fig. 1c) display pronounced revivals of ${{\mathcal{I}}}_{{\bf{z}}}(t)$, consistent with their proximity to KAM-like islands. By contrast, trajectories launched in chaotic regions exhibit rapidly decaying imbalance, indicating fast thermalization and loss of coherence. This sharp dynamical contrast provides a direct quantum analogue of the classical Poincaré section, with imbalance revivals faithfully reflecting the underlying phase-space structure. The close correspondence between simulation and experiment highlights the robustness of imbalance as a scalable proxy for fidelity, whereas the global features of the resulting Poincaré-section-like diagram (Fig. 1c) align closely with TDVP predictions. As shown in Supplementary Section 5 and Supplementary Fig. 6, the structure of the Poincaré section is insensitive to the size of subsystem *A* used to compute ${{\mathcal{I}}}_{{\bf{z}}}(t)$.

The variational manifold constitutes only an approximation to the full quantum dynamics and their discrepancy can be quantified using quantum leakage^17^. The latter is difficult to directly measure; instead, we consider the co-moving imbalance, ${{\mathcal{I}}}_{{\mathrm{CM}}}\equiv {{\mathcal{I}}}_{{\bf{z}}{(t)}}$, where **z**(*t*) is the classical trajectory defined by the TDVP equations of motion. ${{\mathcal{I}}}_{{\mathrm{CM}}}$ evaluates the imbalance of the full quantum state along the TDVP-predicted path, and it should remain close to unity if TDVP faithfully reproduces the dynamics of experimentally accessible observables. The co-moving imbalance map at a fixed time slice is shown in Fig. 2e,f. Its phase-space profile confirms that the TDVP manifold accurately captures the coherent, low-entanglement sector of the evolution featuring KAM-like trajectories that remain confined to ${\mathcal{M}}$; smaller regular islands remain visible but with a reduced co-moving signal, indicating stronger leakage at later times, whereas chaotic trajectories experience more pronounced leakage into higher-entanglement directions outside ${\mathcal{M}}$.

We note there are small discrepancies between the Poincaré section shown in Fig. 1 and that inferred from experimental measurements, particularly near the upper and lower phase-space boundaries (Fig. 2c,d). These deviations are expected because decoherence effects restrict our measurements to much shorter times compared with classical analysis (Fig. 1); hence, we can only resolve orbits with relatively short periods, that is, the largest regular islands. In Supplementary Section 10 and Supplementary Fig. 14, we demonstrate that the agreement between TDVP and experiment can be improved by considering later-time imbalance dynamics. An additional source of discrepancy is the simple form of the shallow variational circuit, which may not capture finer details of quantum dynamics. Although this could be systematically improved by refining the ansatz, our minimal parameterization in equation (2) is sufficient to capture the essential aspects of the global mixed-phase structure.

## Hybrid feedback control

Characterizing mixed phase-space structures in high-dimensional manifolds is challenging: the Poincaré section captures only a low-dimensional slice of the dynamics, rapidly becoming impractical to analyse and losing its visual intuition as the dimension of ${\mathcal{M}}$ grows. To overcome this, we develop a hybrid feedback protocol that combines short quantum evolutions with classical optimization, which allows to directly ‘filter out’ non-thermal trajectories on quantum hardware. Beyond finding such trajectories, this will allow us to verify the robustness of the mixed phase-space structure, as we expect the regular islands to deform smoothly on varying the system’s parameters. In classical mechanics, such structural stability is a defining feature of mixed phase spaces: weak perturbations modify but do not destroy the coexistence of order and chaos.

To steer quantum dynamics towards a regular region of phase space, we use a hybrid quantum–classical optimization scheme inspired by the ScarFinder algorithm^14^. The key idea is that coherent trajectories remain confined within ${\mathcal{M}}$, whereas thermalizing components tend to rapidly escape from it. Thus, short-time evolution $\hat{U}(\Delta t)={e}^{-i\hat{H}\Delta t/\hslash }$ slightly drives the state away from ${\mathcal{M}}$, whereas projection back onto the manifold, ${{\mathcal{P}}}_{{\mathcal{M}}}$, removes the entanglement-generating component and restores coherence. Repeated application of this evolution–projection cycle acts as a dynamical filter:

4 $$\left.| {\psi }^{(n+1)}\right\rangle ={{\mathcal{P}}}_{{\mathcal{M}}}\,\hat{U}(\Delta t)\,\left.| {\psi }^{(n)}\right\rangle ,$$

where ${{\mathcal{P}}}_{{\mathcal{M}}}$ is implemented through feedback optimization. We note that this evolution–projection framework is algorithmically general and not tied to a specific platform. Our implementation takes advantage of its intrinsically hybrid nature: the time evolution step is executed by the quantum processor under the full many-body Hamiltonian, whereas the projection is realized through measurement-based feedback without requiring explicit access to the quantum state. Under iteration, for sufficiently small Δ*t*, regular motion governed by TDVP flow is reinforced, converging towards a self-consistent orbit that minimizes leakage from ${\mathcal{M}}$. Importantly, the protocol can go beyond solving the TDVP equations if we choose a finite time step Δ*t*, which allows us to identify and stabilize trajectories of the full quantum dynamics that remain closest to ${\mathcal{M}}$ (ref. ^14^).

**Fig. 3:**
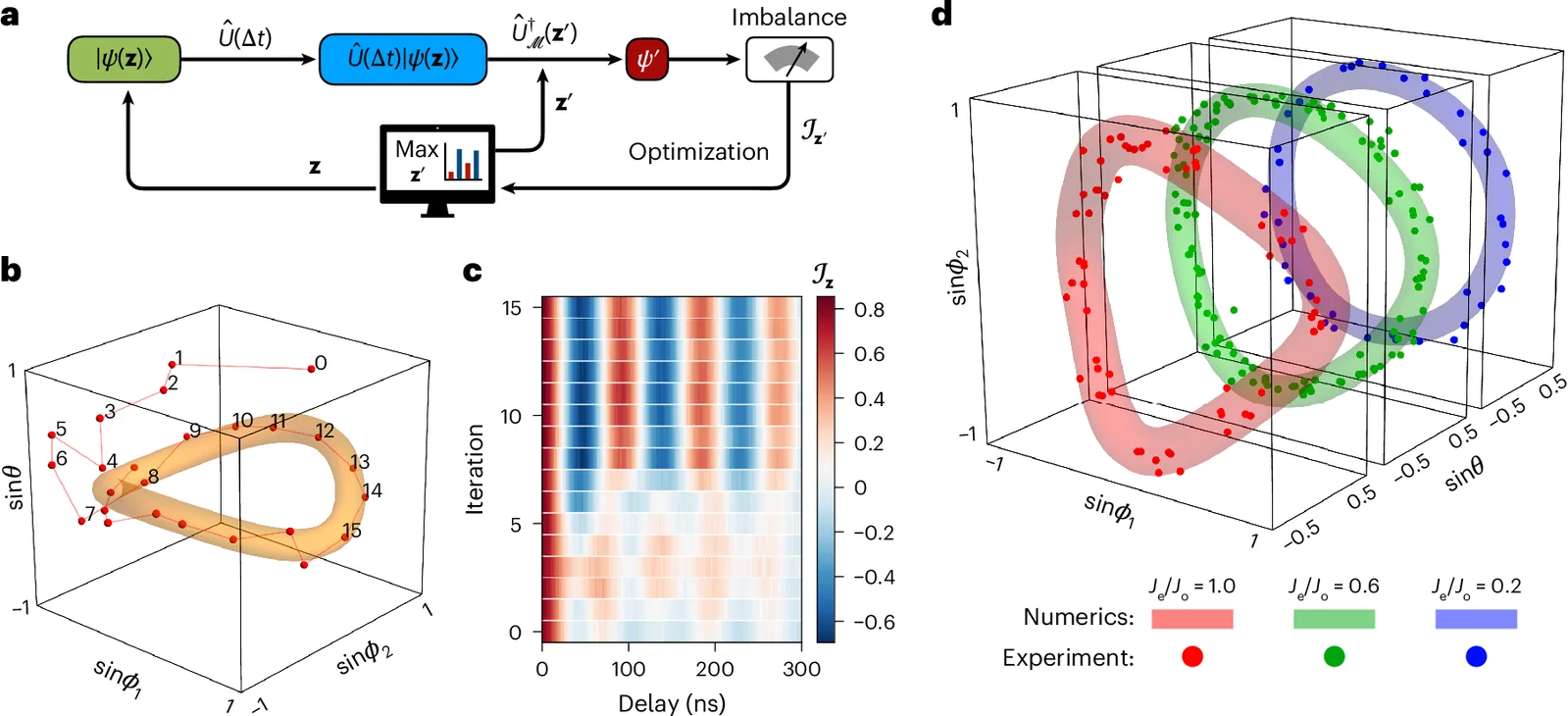
Hybrid feedback control of variational dynamics. **a**, Schematic of the workflow of the iterative feedback loop in equation (4). Starting from an initial state $\left.| \psi ({\bf{z}})\right\rangle$ with parameters (*θ*, *ϕ*_1_, *ϕ*_2_), the system evolves under the unitary $\hat{U}(\Delta t)$ generated by the Hamiltonian in equation (1). An inverse variational circuit ${\hat{U}}_{{\mathcal{M}}}^{\dagger }({{\bf{z}}}^{{\prime} })$ is then applied, and the measured imbalance ${{\mathcal{I}}}_{{{\bf{z}}}^{{\prime} }}$ is maximized through classical optimization, providing updated parameters ${{\bf{z}}}^{{\prime} }$ for the next iteration. **b**, Feedback evolution for a single experimental trajectory on the superconducting processor using 24 qubits, using a time interval of Δ*t* = 80 ns for each feedback step. In each iteration, the imbalance is estimated using 300 independent shots. Starting from **z**^(0)^ = (π/4, 0, π/2), the markers indicate the first few iteration steps, showing the convergence of parameters towards a closed periodic orbit (orange surface) predicted by numerical simulation. **c**, Dynamics of imbalance ${{\mathcal{I}}}_{{\bf{z}}}$ for the 12 central sites for each of the iteration steps marked in **b**, illustrating the steady convergence to the periodic orbit. **d**, Stability of mixed phase space as the even-bond coupling *J*_e_ is varied, with fixed *J*_o_/2π = 5.0 MHz and *J*_3_/2π = 0.25 MHz. Different plots correspond to *J*_e_/2π = 5.0 MHz (front; red), *J*_e_/2π = 3.0 MHz (middle; green) and *J*_e_/2π = 1.0 MHz (back; blue). In each case, the hybrid protocol converged to a stable orbit, whose geometry and curvature vary continuously with *J*_e_, reflecting the smooth deformation of the mixed phase-space structure. Data points are experimental results, whereas the shaded surfaces represent the corresponding stable orbits obtained by a numerical implementation of the protocol. The width of each shaded surface reflects the standard deviation of the experimental data from the numerical prediction. Source data

The experimental workflow of the hybrid feedback procedure is illustrated in Fig. 3a (Methods). Starting from an initial state $\left.| \psi ({{\bf{z}}}^{(0)})\right\rangle$, the system is evolved under the Hamiltonian in equation (1) for a short time Δ*t*. A reverse variational circuit ${\hat{U}}_{{\mathcal{M}}}^{\dagger }({{\bf{z}}}^{{\prime} })$ with adjustable parameters ${{\bf{z}}}^{{\prime} }$ is then applied to this state, followed by the local measurement of imbalance ${{\mathcal{I}}}_{{{\bf{z}}}^{{\prime} }}$. The measured imbalance is processed by a classical optimizer, which updates ${{\bf{z}}}^{{\prime} }$ to maximize ${{\mathcal{I}}}_{{{\bf{z}}}^{{\prime} }}$, producing a refined parameter set **z**^(1)^ that defines the projected state $\left.| \psi ({{\bf{z}}}^{(1)})\right\rangle$ on the variational manifold ${\mathcal{M}}$. This new state is re-prepared on the processor to initiate the next iteration. Repeating this sequence (short-time evolution, inverse circuit, measurement and feedback) generates a discrete trajectory {**z**^(*n*)^} in the variational space. Convergence is achieved when the parameters stabilize, indicating that the feedback loop in equation (4) has locked onto a self-consistent periodic orbit of the hybrid map.

Intuitively, our hybrid loop operates as a closed learning process that ‘distils’ coherence from chaotic motion: each forward evolution step introduces small deviations due to entanglement and experimental noise as each feedback projection realigns the state with the manifold. The resulting steady orbit corresponds to the most regular trajectory accessible within ${\mathcal{M}}$—a dynamically stabilized mode that simultaneously suppresses entropy growth and enhances revival fidelity. Importantly, these trajectories are not generated by the feedback procedure itself, but correspond to dynamical structures already present in the underlying quantum evolution, which the protocol selectively stabilizes and renders experimentally accessible. This self-correcting feedback mechanism makes the hybrid scheme particularly suitable for noisy quantum devices, where short, accurate operations can be repeated even though long, coherent evolution is not achievable. It can be straightforwardly extended to deeper variational circuits by optimizing over additional layers or parameters within the same iterative framework.

We implemented the hybrid quantum–classical feedback protocol on the superconducting processor using 24 qubits (Fig. 3b). We initialize a representative state with parameters (*θ*, *ϕ*_1_, *ϕ*_2_) = (π/4, 0, π/2), residing deep within the chaotic region of the TDVP phase space. After fewer than ten iterations of the hybrid protocol, this trajectory converges to the optimal classical orbit predicted by TDVP (orange surface). We note that the shape of this orbit depends on Δ*t*: a finite Δ*t* introduces a small deformation of the classical trajectory, and this deviation vanishes in the Δ*t* → 0 limit (Supplementary Section 8 and Supplementary Fig. 12). This deformation is intentionally leveraged in the experiment: we adopt Δ*t* = 80 ns, which is close to the system’s intrinsic timescale, *ℏ*/*J*_o_ ≈ 200 ns, to ensure the rapid convergence of the hybrid feedback loop. The dynamics of imbalance ${{\mathcal{I}}}_{{\bf{z}}}(t)$ at each iteration step, measured on the processor using the same method as that in Fig. 2b, are shown in Fig. 3c. These demonstrate that the feedback algorithm reliably converges to a stable trajectory, exhibiting persistent oscillations in ${{\mathcal{I}}}_{{\bf{z}}}(t)$, even in the presence of device noise, crosstalk and calibration imperfections. The discrete parameter trajectories {**z**^(*n*)^}, extracted from successive feedback iterations, gradually approach the periodic orbit, tracing its basin of attraction in parameter space. Small fluctuations around the converged trajectory shown in Fig. 3c are attributed to residual experimental noise rather than instability of the algorithm (Supplementary Section 7 and Supplementary Figs. 9–11).

Finally, in Fig. 3d, we directly probe the stability of the mixed phase space by varying the coupling strength. Fixing *J*_o_/2π = 5.0 MHz and *J*_3_/2π = 0.25 MHz, we run the previously described hybrid protocol for different values of *J*_e_/2π = 1.0, 3.0 and 5.0 MHz. In each case, the feedback dynamics identify a family of stable periodic orbits whose curvature and extent systematically evolve with *J*_e_. This continuous deformation of the orbits reflects the reshaping of the mixed phase-space structure and confirms that the geometry of regular regions can be directly tuned by the underlying Hamiltonian parameters. We emphasize that the same iterative method also provides the means for preparing long-lived non-thermal states, without requiring any input about the model beyond the definition of the manifold ${\mathcal{M}}$.

## Conclusion and outlook

By combining analogue quantum evolution with classical feedback optimization, we demonstrated a hybrid control protocol that uncovers and stabilizes long-lived coherent trajectories in quantum many-body systems. Each feedback cycle requires only short coherent evolutions followed by local measurements and classical optimization, inherently minimizing decoherence and calibration errors and avoiding the need to reconstruct the full many-body wave function. As a proof of principle, we revealed a regime in which regular and chaotic trajectories coexist within the same interacting qubit model, providing experimental evidence for an emergent structure of the mixed phase space in a quantum many-body system.

An important question concerns the many-body nature of the observed mixed phase space. In contrast to conventional few-body systems, the regular islands identified here emerge within a high-dimensional interacting quantum system embedded in a thermalizing background. Consequently, these islands are not dynamically isolated from the thermal sector: rather than exhibiting bounded entanglement, they display slower and more structured entanglement growth than chaotic trajectories (Supplementary Section 9 and Supplementary Fig. 13a). This underscores a key distinction between conventional semiclassical mixed phase space and its many-body counterpart: in the latter case, regular dynamics coexist with entanglement generation and the phase-space structure emerges from the underlying many-body dynamics, in contrast to recent studies of Bose–Hubbard systems that focus on mean-field or few-body regimes^25,26^.

The mixed phase space observed here bears some similarity with the phenomenon of quantum many-body scars, as the latter are also associated with regular dynamics although from a few special initial states^12,13^. A key distinction, however, lies in the structure of trajectories in the space of initial conditions: by analogy with single-particle quantum scars^27^, many-body scarring is associated with isolated unstable periodic orbits, whereas in the present case, regular dynamics are supported by a finite-measure region of nearby trajectories forming KAM-like structures. In this sense, our feedback protocol reveals that some scarred dynamics can be embedded within a larger structure of the mixed phase space of coexisting regular and chaotic trajectories (Fig. 2). Indeed, as shown in Supplementary Section 4, the reduced TDVP equations for a special choice of couplings and initial conditions reproduce the oscillatory trajectories of the ‘rainbow’ quantum many-body scars^28,29^. This calls for a better understanding of the origin of quantum many-body scarring in relation to its single-particle counterpart^17,30–32^ and the phenomenon of mixed phase space reported here.

More broadly, our protocol can be viewed as a generalization of classical chaos control^33,34^, where unstable periodic orbits are stabilized through continuous feedback, to the quantum domain. The core idea of utilizing feedback to amplify ergodicity breaking has intriguing connections with adaptive quantum dynamics^35–38^ and phase transitions induced by measurements^39–42^ and control^43,44^. Moreover, our protocol complements existing optimization strategies for quantum state preparation based on matrix-product-state methods^45–47^, machine learning^48,49^ and adiabatic evolution^50,51^. All of these approaches can be naturally blended with our feedback framework to explore how semiclassical behaviour emerges and can be controlled in broader classes of many-body Hamiltonians, with straightforward extensions to deeper variational circuits and higher dimensions.

## Methods

### Experimental setup

Our experiments are conducted on a two-dimensional flip-chip superconducting quantum processor comprising 125 frequency-tunable transmon qubits, with each adjacent pair coupled via a tunable coupler. A ladder configuration consisting of 24 qubits and 34 couplers (Fig. 1a) is used with precisely calibrated nearest-neighbour coupling strengths of approximately 0–15 MHz to realize the target Hamiltonian in equation (1) (Supplementary Section 1 and Supplementary Figs. 1 and 2 provide a detailed characterization of the device).

To observe the Poincaré section and implement hybrid feedback control, our experimental protocol integrates both digital quantum circuits and analogue quantum simulation. The procedure starts with the execution of shallow quantum circuits, after which all qubits are switched from their individual circuit’s frequency to the resonance frequency at 3.7 GHz. When qubits are brought into resonance at the operating point, the inter-qubit couplings are dynamically activated to implement the desired Hamiltonian evolution with interaction strengths controlled via tunable couplers. Following the evolution periods of the desired time, the qubits are returned to their idle frequencies to execute the reversed gate sequence. Throughout these reference frame transformations, all qubits accumulate additional phases that are compensated in our calibration protocol (Supplementary Section 2).

The processor features capacitively coupled read-out resonators designed at around 6.4 GHz for dispersive measurement, enabling simultaneous qubit-state detection with average fidelity exceeding 98%. The system is integrated using wire-bonding techniques and protected by multilayer magnetic shields in a dilution refrigerator operating at 15 mK.

### Shallow circuit implementation

The shallow circuit is implemented by compiling the required XY gates as a combination of single-qubit gates and CZ gates as follows:Single-qubit gates are implemented using 20-ns microwave pulses with a Gaussian envelope, optimized via the derivative reduction by adiabatic gate technique. The CZ gates are realized by tuning $\left.| 11\right\rangle$ and $\left.| 20\right\rangle$ states of the two qubits near resonance and switching on the coupling by applying a fast flux pulse to the corresponding coupler between them for a duration of 32 ns. Benchmarking with simultaneous cross-entropy benchmarking protocols reveals single-qubit gate fidelities exceeding 99.95% and two-qubit CZ gate fidelities above 99.5%. Additionally, arbitrary single-qubit rotations are decomposed into a virtual Z-phase gate followed by an actual XY rotation. The details of the fidelity of our gates are shown in Supplementary Section 1 and Supplementary Fig. 2.

### Equations of motion for the shallow circuit ansatz

By projecting quantum dynamics generated by the Hamiltonian in equation (1) onto the variational manifold spanned by states in equation (2), we obtain the following TDVP equations of motion:

5 $$\begin{array}{l}\dot{\theta }={J}_{\mathrm{e}}\cos {\phi }_{1}\cos {\phi }_{2}-{J}_{3}{\cos }^{2}(2\theta )\,\sin {\phi }_{1}\sin {\phi }_{2},\\ {\dot{\phi }}_{1}={J}_{\mathrm{o}}+\displaystyle\frac{\sin (4\theta )\,\left({J}_{\mathrm{e}}\sin {\phi }_{1}\cos {\phi }_{2}+{J}_{3}\sin {\phi }_{2}\cos {\phi }_{1}\right)}{1+{\cos }^{2}(2\theta )},\\ {\dot{\phi }}_{2}={J}_{\mathrm{o}}+\displaystyle\frac{\sin (4\theta )\,\left({J}_{\mathrm{e}}\sin {\phi }_{2}\cos {\phi }_{1}+{J}_{3}\sin {\phi }_{1}\cos {\phi }_{2}\right)}{1+{\cos }^{2}(2\theta )},\end{array}$$

Supplementary Section 4 provides the full derivation. By integrating these equations, we obtain the phase-space portrait shown in Fig. 1d. In contrast to the underlying linear Schrödinger equation, the projected dynamics generated by equation (5) are evidently nonlinear. On one hand, this nonlinearity allows for the emergence of a mixed phase space, whereas on the other hand, it implies that the TDVP evolution cannot perfectly reproduce the exact quantum dynamics. As we enlarge the variational manifold, we can represent increasingly finer details of the full quantum dynamics. With this caveat in mind, our variational ansatz is the minimal description that captures the essential dynamical features of the SSH ladder in an analytically tractable way, illustrating the power of this approach.

### Experimental measurement of fidelity

Fidelity provides a natural measure of how closely a quantum system returns to its initial state under time evolution:

6 $$F(t)=| \langle \psi (0)| \psi (t)\rangle {| }^{2}.$$

In general, extracting *F*(*t*) in a many-body system requires full quantum state tomography, which is exponentially costly in terms of system size and, therefore, impractical for our quantum processor.

In our setting, however, the fidelity can be accessed more efficiently using a Loschmidt-echo-type protocol. The initial state $\left.| \psi (0)\right\rangle$ is prepared by a known unitary circuit ${\hat{U}}_{{\mathcal{M}}}$ acting on a product state $\left\vert 0101\ldots \right\rangle$. After evolving the system for a time *t* under $\hat{U}(t)$, we apply the inverse circuit ${\hat{U}}_{{\mathcal{M}}}^{\dagger }$. This maps the overlap onto a measurement probability:

7 $$F(t)=\Pr \left[\,\mathrm{measure}\,\left\vert 0101\ldots \right\rangle \,\mathrm{after}\,{\hat{U}}_{\mathcal{M}}^{\dagger}\hat{U}(t)\right],$$

which counts how often the reference computational basis state is observed in bitstring snapshots.

However, for a 24-qubit system, the reference bitstring typically appears with exponentially small probability, far below the experimental noise floor. To obtain a robust signal, we therefore introduce the subsystem fidelity, defined by checking whether a chosen block of *L*_sub_ consecutive qubits matches the reference pattern. For each snapshot, the subsystem fidelity contribution is one if the block matches and zero otherwise; averaging over all shots yields

8 $${F}_{\mathrm{sub}}(t)=\Pr\Biggl[{\mathrm{measure} }\;|\ldots{\mathop{\underbrace{{0101\ldots01}}}\limits_{{L}_{\mathrm{sub}}}}\ldots\rangle \;{\mathrm{at}}\;{\mathrm{time}}\;t\Biggr].$$

Because only *L*_sub_ spins are compared, *F*_sub_(*t*) remains experimentally accessible even for moderate subsystem sizes.

Extended Data Fig. 1a shows *F*_sub_(*t*) for several subsystem sizes, extracted for the initial condition (*θ*, *ϕ*_2_) = (0.05π, 0.5π). Smaller subsystems produce smoother, less noisy behaviour, whereas larger subsystems more closely approximate the global fidelity but exhibit stronger statistical fluctuations. For all choices of *L*_sub_, the revival structure follows that of the imbalance ${{\mathcal{I}}}_{{\bf{z}}}(t)$, demonstrating that both observables encode essentially the same dynamical information.

To visualize the dependence on initial conditions, we construct a Poincaré-section-like map using the first revival peak ${F}_{{\rm{sub}}}^{\max }$ for each (*θ*, *ϕ*_2_) (Extended Data Fig. 1b). Despite increased noise and reduced contrast compared with the imbalance-based map ${{\mathcal{I}}}_{{\bf{z}}}^{\max }$ used in the main text, the qualitative phase-space structure is reproduced.

In summary, imbalance and fidelity diagnose regular versus chaotic dynamics in the same qualitative manner. The imbalance, however, is simpler and yields higher-contrast maps across the full phase space. For this reason, we rely on imbalance as the primary experimental observable in the main text, whereas fidelity-based measurements provide supporting validation of the results.

### Algorithmic structure and physical interpretation of the hybrid protocol

Our hybrid optimization protocol is implemented as an iterative feedback loop that alternates between short quantum evolutions and classical parameter updates. Each iteration approximates a discrete TDVP step, with the projection onto the variational manifold realized experimentally through measurement and optimization rather than analytically.

We begin by preparing a state $\left.| \psi ({{\bf{z}}}^{(0)})\right\rangle$ with parameters ${{\bf{z}}}^{(0)}=({\theta }^{(0)},{\phi }_{1}^{(0)},{\phi }_{2}^{(0)})$ (equation (2)) on a quantum processor. We then iterate through the following sequence:

1. Time evolution: evolve for a short interval Δ*t*, $\left.| \psi \right\rangle =\hat{U}(\Delta t)\left.| \psi ({{\bf{z}}}^{(0)})\right\rangle$, with the unitary $\hat{U}(\Delta t)={e}^{-i\hat{H}\Delta t/\hslash }$ corresponding to the Hamiltonian $\hat{H}$.
2. Reverse circuit: apply the inverse variational circuit ${\hat{U}}_{{\mathcal{M}}}^{\dagger }({{\bf{z}}}^{{\prime} })$ (equation (2)), parameterized by a trial set of variables ${{\bf{z}}}^{{\prime} }$, to obtain $\left.| {\psi }^{{\prime} }\right\rangle ={\hat{U}}_{{\mathcal{M}}}^{\dagger }({{\bf{z}}}^{{\prime} })\left.| \psi \right\rangle$.
3. Measurement: measure the local spin expectations $\langle {\hat{\sigma }}_{j}^{z}\rangle$ and compute the imbalance ${{\mathcal{I}}}_{{{\bf{z}}}^{{\prime} }}$ (equation (3)).
4. Classical optimization: the measured imbalance is processed by a classical optimizer, which updates the parameters ${{\bf{z}}}^{{\prime} }$ to maximize the overlap with the evolved state. The optimal parameters, ${{\bf{z}}}^{(1)}={\arg \max }_{{{\bf{z}}}^{{\prime} }}\,{{\mathcal{I}}}_{{{\bf{z}}}^{{\prime} }}[\left.| \psi \right\rangle ]$, define the best variational approximation $\left.| \psi ({{\bf{z}}}^{(1)})\right\rangle$ to the evolved state $\left.| \psi \right\rangle$.
5. Reinitialization: prepare the updated state $\left.| \psi \right\rangle =\left.| \psi ({{\bf{z}}}^{(1)})\right\rangle$ and repeat the loop.

Experimentally, each function evaluation in step 4 above corresponds to a complete quantum measurement. Typical convergence for the next evolved state requires 50–200 iterations, with the total experimental duration spanning approximately 0.5–2 h. To mitigate finite-size boundary effects, we use the imbalance (measured at Δ*t* = 80 ns) of the four central qubits as the observable for optimizing the parameters **z**^(*n*)^. The resultant optimal set is then used to reinitialize and reprepare the quantum state for subsequent measurement cycles. Iterating this feedback cycle generates a discrete trajectory {**z**^(*n*)^} within the variational manifold ${\mathcal{M}}$. Convergence is reached when the parameters stabilize along a closed orbit (typically within a few hundreds of iterations), indicating that the feedback loop has identified a self-consistent periodic trajectory under evolution and projection, which is the most stable dynamical mode supported by the manifold.

## Online content

Any methods, additional references, Nature Portfolio reporting summaries, source data, extended data, supplementary information, acknowledgements, peer review information; details of author contributions and competing interests; and statements of data and code availability are available at https://doi.org/10.1038/s41567-026-03431-z.

## Supplementary information

Supplemental Information Supplementary Sections 1–11 (describing the device performance, hybrid quantum simulation protocol, alternative optimization scheme, TDVP derivation for the shallow circuit ansatz, Poincaré sections for different couplings and under perturbations, finite-size scaling, realistic modelling of the experiment, convergence of the hybrid protocol, mixed phase space beyond local observables, refined Poincaré section analysis and numerical methods), Figs. 1–14, Table 1 and references.

## Source data

Source Data Fig. 1 Statistical source data including TDVP trajectories and TDVP Poincaré section points.

Source Data Fig. 2 Statistical source data including imbalance time series, first-revival-peak data and co-moving imbalance data.

Source Data Fig. 3 Statistical source data including hybrid feedback trajectory, imbalance per iteration and stable orbits versus coupling.

Source Data Extended Data Fig. 1 Statistical source data including subsystem fidelity time series and fidelity revival data.

## Data Availability

The data that support the findings of this study are openly available via the University of Leeds Research Data Repository at https://doi.org/10.5518/1890 (ref. ^52^). Source data are provided with this paper.

## Code availability

The numerical calculations were performed using the open-source Julia packages EDKit.jl and InfiniteTEBD.jl, available via GitHub at https://github.com/jayren3996.
