# Supplementary material for: Quantum many-body mixed phase space revealed by hybrid feedback control
Source: Nat Phys. 2026 Sep 23;22(11):1754–60. doi: 10.1038/s41567-026-03431-z (PMC13759323; doi:10.1038/s41567-026-03431-z)
Supplement: Supplementary file 1 [file 41567_2026_3431_MOESM1_ESM.pdf]

nature physics

Supplementary information

https://doi.org/10.1038/s41567-026-03431-z

Quantum many-body mixed phase space revealed by hybrid feedback control

In the format provided by the authors and unedited

CONTENTS

S1. Device performance 1

S2. Hybrid quantum simulation 1

S3. Alternative optimization scheme 4

S4. Time-dependent variational principle for shallow circuit ansatz 5
A. Gram matrix 6
B. Hamiltonian gradients 7
C. Equations of motion 8
D. Exact rainbow scar point 9

S5. Poincaré sections for different couplings and under perturbations 9

S6. Finite-size scaling of the Poincaré section 12

S7. Realistic modeling of the experiment 12

S8. Convergence of the hybrid protocol 15

S9. Quantum many-body mixed phase space beyond local observables: inverse participation ratio, entanglement and PCA entropy 16

S10. Refined Poincaré section analysis 18

S11. Numerical methods 19
A. Exact diagonalization 19
B. Matrix-product-state methods 20
C. Hybrid feedback map 20

References 21

S1. DEVICE PERFORMANCE

The wiring information and room temperature control electronics are similar to those of [1]. Figure S1 summarizes the typical single-qubit parameters of our processor, where single-qubit gates are applied at idle frequencies ranging from 3.5 GHz to 3.96 GHz. The energy relaxation time T_1 and dephasing time T_2 measured at these idle frequencies are also reported; both are substantially longer than the execution time of our shallow circuits. To enhance readout fidelity, an additional microwave pulse is applied to each qubit prior to the readout pulse, driving the $|1\rangle \leftrightarrow |2\rangle$ transition. The readout error, defined as the average misassignment probability for the $0\rangle$ and $1\rangle$ states, is predominantly below 1% across all qubits. All data presented in this work have been processed with readout error mitigation.

As detailed in the Methods section, our experimental sequence involves compiling shallow circuits into combinations of single-qubit gates and two-qubit CZ gates to prepare the targeted states. During this process, all qubits are maintained at their idle frequencies, while the couplers are kept in a nearly off state. To minimize spurious interactions, we actively control not only the qubits forming the ladder geometry but also two adjacent rows of surrounding qubits and all associated couplers. Consecutive single-qubit gates are consolidated into a single XY microwave rotation combined with a virtual Z phase adjustment, thereby reducing the total number of physical gate operations. We characterize gate performance via simultaneous cross-entropy benchmarking (XEB), achieving median fidelities of approximately 99.95% for single-qubit gates and 99.5% for two-qubit gates, respectively (Fig. S2).

S2. HYBRID QUANTUM SIMULATION

Our experimental protocol integrates digital quantum circuits with analog quantum simulation. The procedure begins with the execution of shallow quantum circuits, after which all qubits are switched from the gate-operation reference frame to the Hamiltonian evolution reference frame. When the qubits are tuned into resonance, the

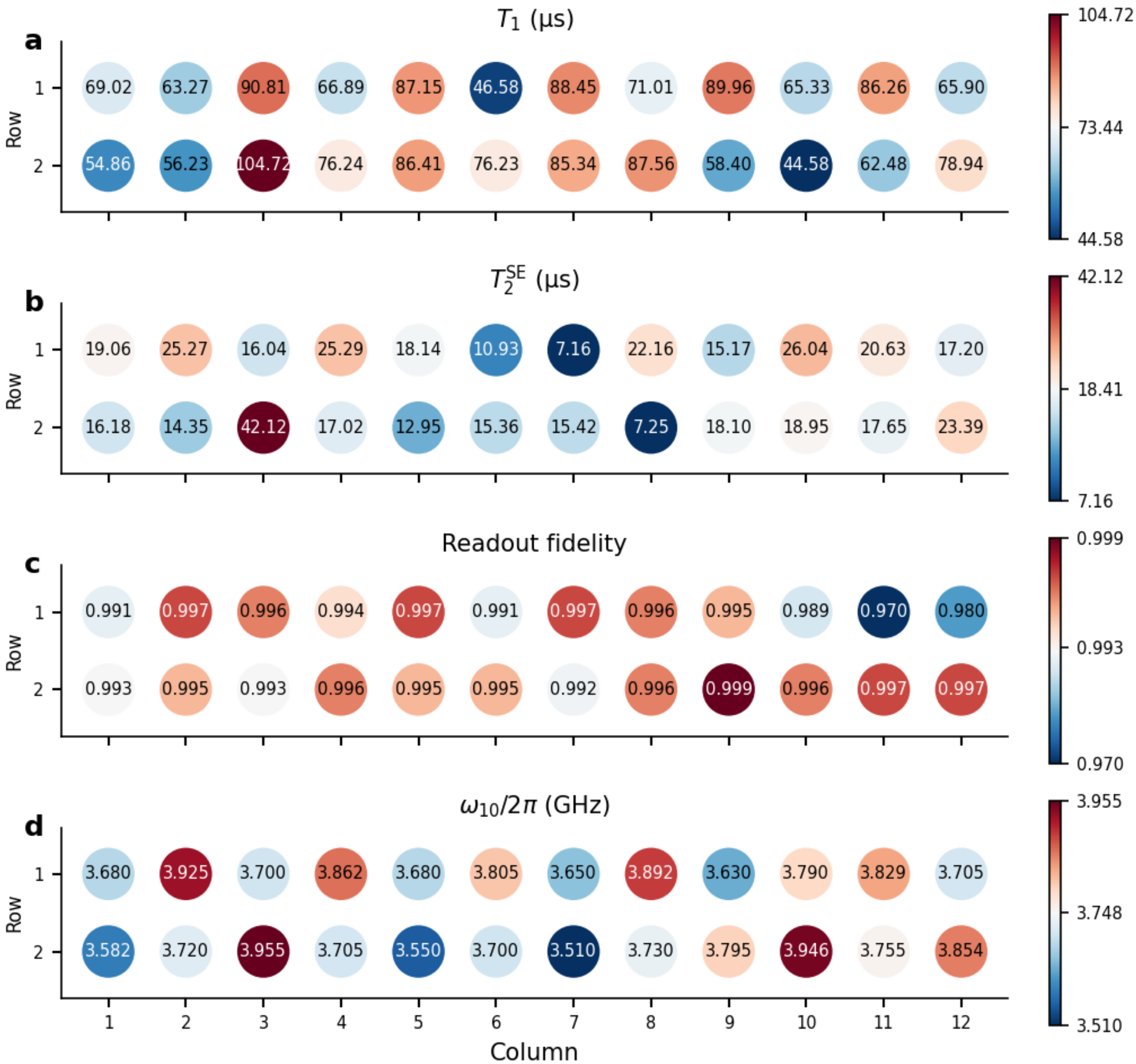

Figure S1. **Heat map of single-qubit parameters.** Top to bottom: Qubit relaxation time measured at its idle frequency, with 73.62 μs the median for all qubits; Qubit dephasing time measured using Hahn echo sequence at its idle frequency, with 17.4 μs the median for 24 qubits; Readout error averaged for qubit in $|0\rangle$ and $|1\rangle$, measured by preparing random product states on all qubits, with the median error rate 0.76%; Qubit idle frequency.

inter-qubit couplings are activated to realize the target Hamiltonian evolution. After evolution, the qubits are shifted from the resonance frequency of 3.7 GHz back to their respective idle frequencies to implement the reversed quantum gate sequence. Throughout this reference frame transformation, all qubits accumulate additional phases.

To calibrate and compensate for these phase shifts via single-qubit gates, we implement the following calibration procedure:

1. We first coarsely adjust all coupling strengths to match the target Hamiltonian parameters. To calibrate the resonance condition, we perform swap spectroscopy between each pair of qubits while detuning all other qubits by at least 100 MHz from the interaction band. This ensures precise resonance alignment across the qubit array.

2. For each individual qubit, we perform quantum state tomography after Hamiltonian evolution under the required coupling strength of the couplers, while maintaining all other qubits detuned by approximately ± 100 MHz from their

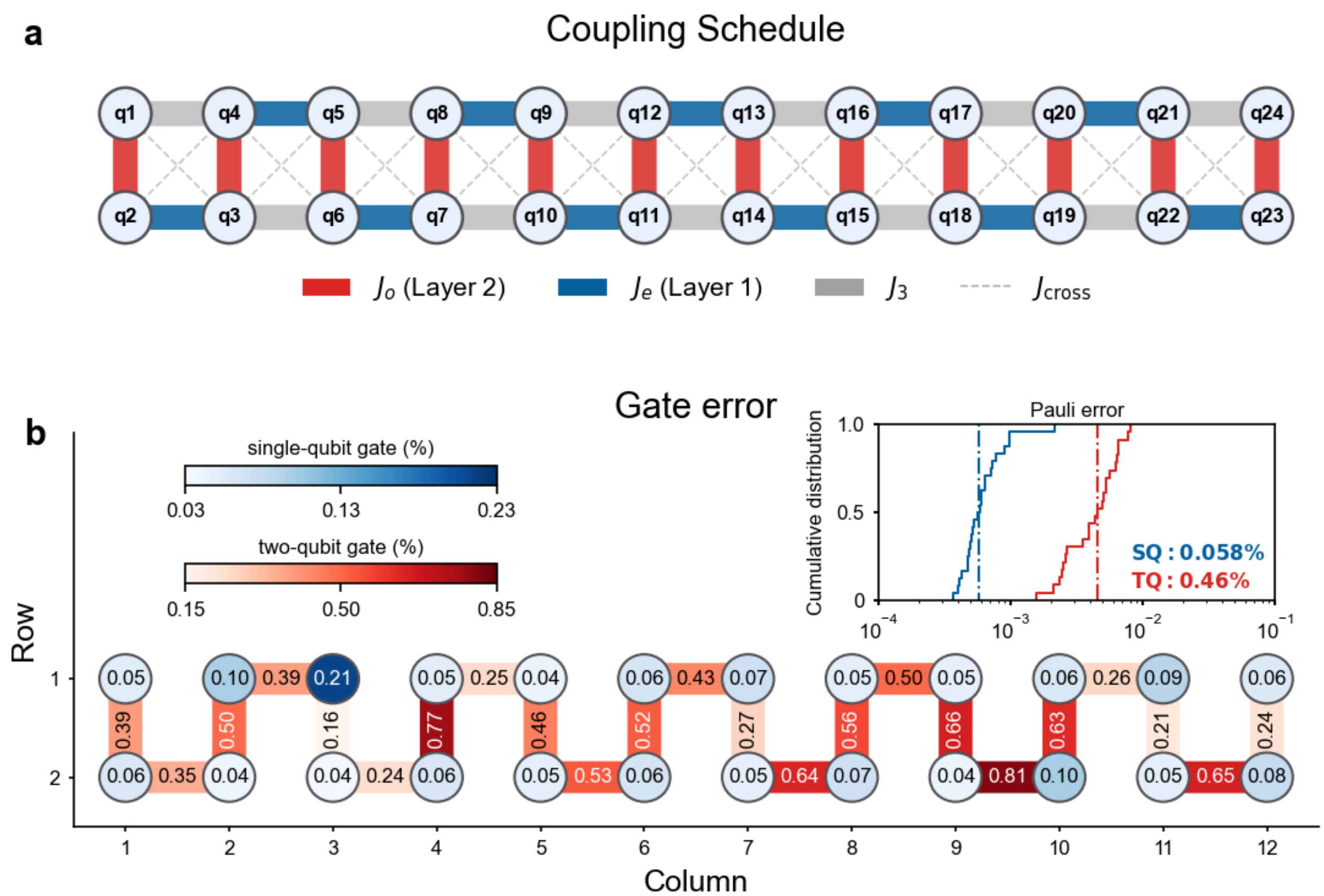

Figure S2. **Coupling schedule and Pauli errors of both single-qubit and two-qubit gates. a**, Coupling structure of the device. Qubits are numbered following the same order as in Figure S1. The CZ gates are implemented in two layers: J_e couplers are activated simultaneously in Layer 1, and J_o couplers are activated simultaneously in Layer 2. **b**, Gate errors are benchmarked with simultaneous cross-entropy benchmarking (XEB). Errors of single-qubit gates (blue circles) are obtained by running single-qubit XEB sequences for all qubits simultaneously, while errors of two-qubit gates (red bars) are averaged over the two-qubit layers used in our experiments. For each two-qubit layer, we run two-qubit XEB sequences simultaneously for all the two-qubit gates in this layer. The inset shows the cumulative distribution of gate errors, with the dashed lines indicating the median values.

resonant frequencies. This configuration enables accurate experimental tracking of phase accumulation induced by reference frame transitions. We then finely adjust the Z-pulse amplitude (zpa) for each qubit until the accumulated phase satisfies the relation:

$$\Delta\phi = (\omega_{\text{resonance}} - \omega_{\text{gates}}) \times t\,. \tag{S1}$$

3. The additional phase for the ith qubit is calculated as $(\omega^i_{\text{resonance}} - \omega^i_{\text{gates}}) \times t_{\text{circuit}}$, where $t_{\text{circuit}} = 228$ ns. This phase correction is applied via single-qubit Z-rotations both before and after the analog evolution segment. Since our gate sequence consists of a layer of single-qubit gates followed by a layer of two-qubit gates, this phase correction can be efficiently implemented via virtual Z gates—that is, by adjusting the phase of subsequent XY pulses—rather than applying physical Z rotations.

4. Finally, we characterize and compensate for residual relative global phases between all qubit pairs in a sequential chain from the first to the last qubit, by introducing corresponding initial phase offsets in subsequent XY rotation pulses.

S3. ALTERNATIVE OPTIMIZATION SCHEME

In the main text, the imbalance observable $\mathcal{I}_{\boldsymbol{z}}$ was measured by applying the inverse circuit $\hat{U}^{\dagger}_{\mathcal{M}}(\boldsymbol{z})$ before readout:

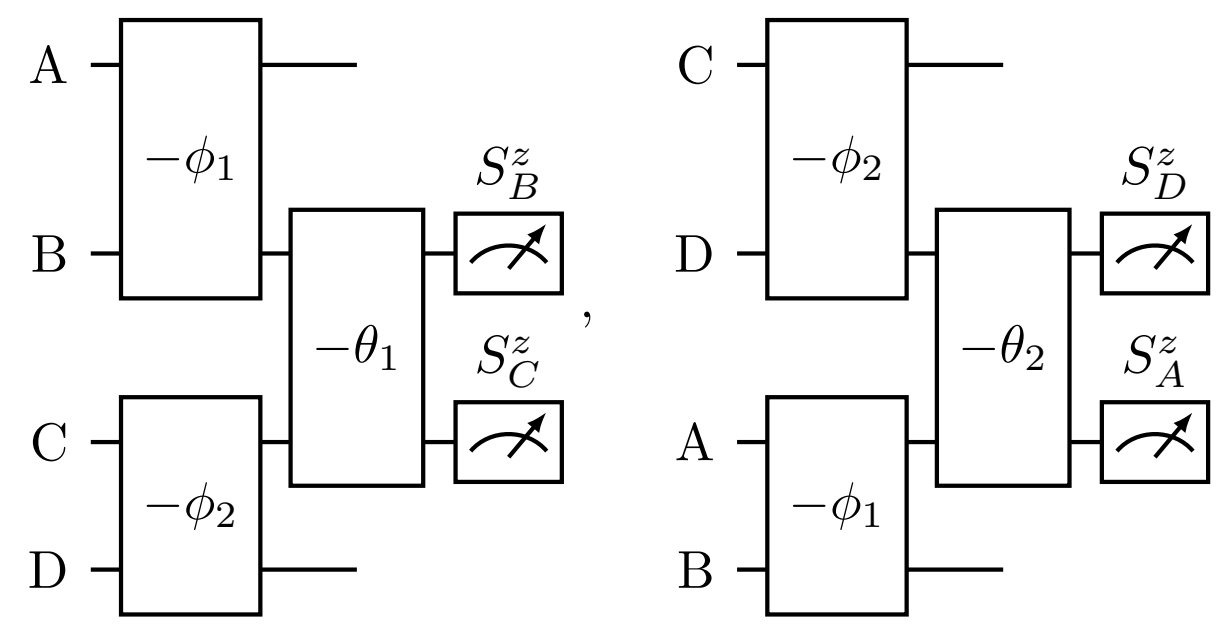

This protocol directly probes the overlap between the evolved state and the variational ansatz parametrized by $\boldsymbol{z}$, and thus provides a practical proxy for the state fidelity. However, in practice, scanning the imbalance over many parameter choices $\boldsymbol{z}$ requires separate state preparation and reverse-gate operation for each setting, which quickly becomes resource-intensive. To mitigate this overhead, it is desirable to have an alternative scheme in which all measurements are performed in a fixed basis, while the results for arbitrary $\boldsymbol{z}$ can be reconstructed from a common set of measurements with analytically known coefficients. Such a scheme can be obtained by carrying out the evolution of an observable in the Heisenberg picture rather than evolving the state.

Specifically, we consider

$$\begin{aligned}\hat{O}_1(\theta_1,\phi_1,\phi_2) &= U^{\dagger}(\theta_1,\phi_1,\phi_2)(S_B^z - S_C^z)U(\theta_1,\phi_1,\phi_2),\\ \hat{O}_2(\theta_2,\phi_1,\phi_2) &= U^{\dagger}(\theta_2,\phi_1,\phi_2)(S_D^z - S_A^z)U(\theta_2,\phi_1,\phi_2),\end{aligned} \tag{S2}$$

whose dependence on $\boldsymbol{z}$ can be determined analytically within the variational manifold. Once this operator is expressed as a linear combination of a fixed set of local correlators, the imbalance for any $\boldsymbol{z}$ follows from a weighted sum of those correlators, all measurable within a single experimental configuration. In the following, we derive the explicit form of the rotated operators $\tilde{\sigma}_j^z(\boldsymbol{z})$ and show how the resulting linear combinations reproduce the imbalance landscape without requiring separate state re-preparation for each parameter value.

The explicit form of the observables are:

$$\begin{aligned}\hat{O}_1(\theta_1,\phi_1,\phi_2) =\ & \cos(2\theta_1)\left(\sin^2\phi_1 Z_A + \cos^2\phi_1 Z_B - \cos^2\phi_2 Z_C - \sin^2\phi_2 Z_D\right)\\ & + \cos(2\theta_1)\left[\sin(2\phi_1)X_AY_B + \sin(2\phi_2)X_CY_D\right]\\ & - 2\sin(2\theta_1)\left(\cos\phi_1\cos\phi_2 X_BY_C + \sin\phi_1\sin\phi_2 X_AZ_BZ_CY_D\right)\\ & - 2\sin(2\theta_1)\left(\sin\phi_1\cos\phi_2 X_AZ_BX_C - \sin\phi_2\cos\phi_1 X_BZ_CX_D\right).\\ \hat{O}_2(\theta_2,\phi_1,\phi_2) =\ & \cos(2\theta_2)\left(\sin^2\phi_2 Z_C + \cos^2\phi_2 Z_D - \cos^2\phi_1 Z_A - \sin^2\phi_1 Z_B\right)\\ & + \cos(2\theta_2)\left[\sin(2\phi_2)X_CY_D + \sin(2\phi_1)X_AY_B\right]\\ & - 2\sin(2\theta_2)\left(\cos\phi_1\cos\phi_2 X_DY_A + \sin\phi_1\sin\phi_2 X_CZ_DZ_AY_B\right)\\ & - 2\sin(2\theta_2)\left(\sin\phi_2\cos\phi_1 X_CZ_DX_A - \sin\phi_1\cos\phi_2 X_DZ_AX_B\right).\end{aligned} \tag{S3}$$

Note that we have used the symmetric definition for operators X and Y: X_iX_j pair always means $(X_iX_j + Y_iY_j)/2$, and X_iY_j pair always means $(X_iY_j - Y_iX_j)/2$. When $\theta_1 = \theta_2 = \theta$, the sum of two operators is:

$$\begin{aligned}[\hat{O}_1 + \hat{O}_2](\theta,\phi_1,\phi_2) =\ & -\cos(2\theta)\left[\cos(2\phi_1)(Z_A - Z_B) + \cos(2\phi_2)(Z_C - Z_D)\right]\\ & + 2\cos(2\theta)\left[\sin(2\phi_1)X_AY_B + \sin(2\phi_2)X_CY_D\right]\\ & - 2\sin(2\theta)\cos\phi_1\cos\phi_2(X_BY_C + X_DY_A)\\ & - 2\sin(2\theta)\sin\phi_1\sin\phi_2(X_AZ_BZ_CY_D + X_CZ_DZ_AY_B)\\ & + 2\sin(2\theta)\sin\phi_1\cos\phi_2(X_DZ_AX_B - X_AZ_BX_C)\\ & + 2\sin(2\theta)\sin\phi_2\cos\phi_1(X_BZ_CX_D - X_CZ_DX_A).\end{aligned} \tag{S4}$$

These two local observables are combinations of fixed local operators, with specific coefficients depending on θ, ϕ.

Experimentally, given a quantum state, the variational parameters of the ansatz can be inferred by maximizing the expectation value of suitably chosen local observables. In particular,

$$\{\theta, \phi_1, \phi_2\} = \underset{\theta,\phi_1,\phi_2}{\operatorname{argmax}} \left[\langle \hat{O}_1 + \hat{O}_2 \rangle (\theta, \phi_1, \phi_2) \right], \tag{S5}$$

where $\hat{O}_{1,2}$ denote experimentally accessible operators such as local imbalances or spin correlators.

This procedure plays a key role in the hybrid feedback protocol: it transforms the quantum-classical optimization step into a purely classical optimization problem. Rather than reconstructing the full quantum state multiple times, one only measures the expectation values of a few local observables and uses their classical post-processing to update the parameters (θ, ϕ_1, ϕ_2). Consequently, the feedback loop alternates between short quantum evolutions and entirely classical optimization steps, minimizing experimental overhead while retaining full access to the relevant variational degrees of freedom.

S4. TIME-DEPENDENT VARIATIONAL PRINCIPLE FOR SHALLOW CIRCUIT ANSATZ

We begin by constructing a variational ansatz based on nearest-neighbor XY rotations applied to the Néel-ordered product state $|\mathbb{Z}_2\rangle \equiv |01\ldots01\rangle$, which serves as a simple unentangled reference with alternating spin order. The ansatz captures the essential entangling dynamics of the interacting SSH ladder through layers of XY rotation gates, each of which acts on a pair of qubits and preserves total magnetization. The two-qubit rotation is defined as

$$\mathrm{XY}(\theta) = \begin{bmatrix} 1 & 0 & 0 & 0 \\ 0 & \cos\theta & i\sin\theta & 0 \\ 0 & i\sin\theta & \cos\theta & 0 \\ 0 & 0 & 0 & 1 \end{bmatrix}, \tag{S6}$$

which generates coherent oscillations in the single-excitation subspace $\{|01\rangle, |10\rangle\}$. Applying layers of such gates with alternating parameters produces the variational state

$$|\psi(\theta_1,\theta_2,\phi_1,\phi_2)\rangle = \left[\prod_j \mathrm{XY}_{4j+1,4j+2}(\phi_1)\ \mathrm{XY}_{4j+3,4j+4}(\phi_2)\right] \times \left[\prod_j \mathrm{XY}_{4j,4j+1}(\theta_1)\ \mathrm{XY}_{4j+2,4j+3}(\theta_2)\right] |\mathbb{Z}_2\rangle. \tag{S7}$$

This ansatz admits a natural matrix-product state (MPS) representation. A schematic tensor-network depiction is:

$|\psi\rangle =$ 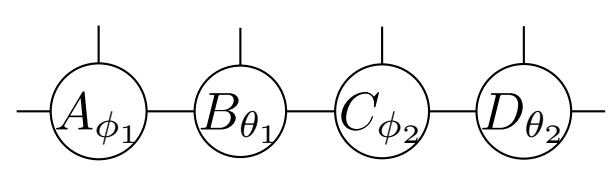 . (S8)

Each tensor is parametrized explicitly by the gate angles:

$$A^{[0]}_{\phi_1} = \begin{bmatrix} 0 & 0 & i\sin(\phi_1) & 0 \\ \cos^2\left(\frac{\phi_1}{2}\right) & \sin^2\left(\frac{\phi_1}{2}\right) & 0 & 0 \end{bmatrix}, \quad A^{[1]}_{\phi_1} = \begin{bmatrix} \cos^2\left(\frac{\phi_1}{2}\right) & -\sin^2\left(\frac{\phi_1}{2}\right) & 0 & 0 \\ 0 & 0 & 0 & i\sin(\phi_1) \end{bmatrix},$$
$$B^{[0]}_{\theta_1} = \begin{bmatrix} \cos(\theta_1) & 0 \\ \cos(\theta_1) & 0 \\ 0 & 0 \\ 0 & i\sin(\theta_1) \end{bmatrix}, \quad B^{[1]}_{\theta_1} = \begin{bmatrix} 0 & i\sin(\theta_1) \\ 0 & -i\sin(\theta_1) \\ \cos(\theta_1) & 0 \\ 0 & 0 \end{bmatrix}, \tag{S9}$$
$$C^{[i]}_{\phi_2} = A^{[i]}_{\phi_1\to\phi_2}, \quad D^{[i]}_{\theta_2} = B^{[i]}_{\theta_1\to\theta_2}.$$

Expectation values and overlaps can be evaluated using the transfer matrices formed by contracting each tensor with its conjugate:

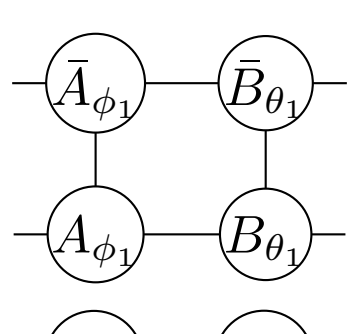 $= \begin{bmatrix} \cos^2\theta_1 & 0 & 0 & \sin^2\theta_1 \\ 0 & 0 & 0 & 0 \\ 0 & 0 & 0 & 0 \\ \cos^2\theta_1 & 0 & 0 & \sin^2\theta_1 \end{bmatrix} =$ 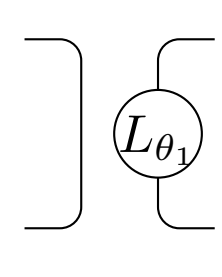 ,

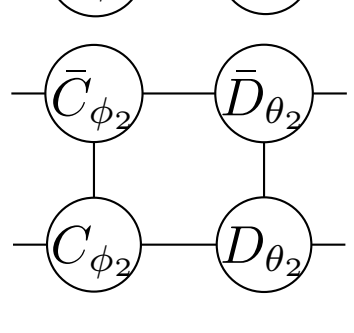 $= \begin{bmatrix} \cos^2\theta_2 & 0 & 0 & \sin^2\theta_2 \\ 0 & 0 & 0 & 0 \\ 0 & 0 & 0 & 0 \\ \cos^2\theta_2 & 0 & 0 & \sin^2\theta_2 \end{bmatrix} =$ 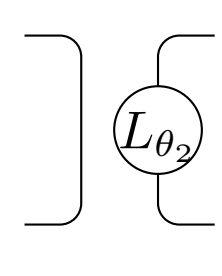 . (S10)

The corresponding left dominant eigenvector is:

$$L_\theta = \begin{bmatrix} \cos^2\theta & 0 \\ 0 & \sin^2\theta \end{bmatrix}. \tag{S11}$$

Time-dependent variational principle (TDVP) provides a systematic way to approximate the real-time evolution of a quantum state by restricting the dynamics to a chosen variational manifold. Concretely, the Schrödinger equation

$$i\frac{\partial}{\partial t}|\psi(\boldsymbol{z})\rangle = \hat{H}|\psi(\boldsymbol{z})\rangle \tag{S12}$$

is projected onto the tangent space of the manifold spanned by the variational parameters $\boldsymbol{z} = \{\theta_1, \theta_2, \phi_1, \phi_2\}$. This projection ensures that the time evolution of the parameters follows the trajectory within the manifold that minimizes the instantaneous distance between the exact and variational time derivatives of the state. As a result, the linear quantum dynamics are transformed into a set of nonlinear coupled equations of motion for the parameters, which can display rich dynamical features including periodic motion, instability, and chaos.

In general, the TDVP equations of motion take the form

$$\sum_l G_{kl}(\boldsymbol{z})\,\dot{z}_l = -i\,h_k(\boldsymbol{z}), \tag{S13}$$

where the Gram matrix is defined as

$$G_{kl}(\boldsymbol{z}) = \langle \partial_{z_k}\psi(\boldsymbol{z})\,|\,\partial_{z_l}\psi(\boldsymbol{z})\rangle, \tag{S14}$$

and the Hamiltonian gradient is given by

$$h_k(\boldsymbol{z}) = \langle \partial_{z_k}\psi(\boldsymbol{z})\,|\,\hat{H}\,|\,\psi(\boldsymbol{z})\rangle. \tag{S15}$$

The expression (S13) represents a system of linear equations for the parameter velocities $\dot{z}_l$, with coefficients determined entirely by the geometry of the variational manifold and the action of the Hamiltonian. In the following, we derive the explicit form of these equations for the XY-rotation ansatz introduced above. We begin by computing the Gram matrix and Hamiltonian gradient, before arriving at closed-form expressions for the equations of motion of the variational parameters $(\theta_1, \theta_2, \phi_1, \phi_2)$.

A. Gram matrix

The Gram matrix encodes the overlaps of tangent vectors $\{|\partial_{z_\mu}\psi\rangle\}$ on the variational manifold,

$$G_{\mu\nu} \equiv \langle \partial_{z_\mu}\psi|\partial_{z_\nu}\psi\rangle, \qquad z_\mu \in \{\theta_1, \theta_2, \phi_1, \phi_2\}.$$

Throughout this section, the prime symbol on a tensor denotes the derivative with respect to the corresponding parameter (e.g., $A' \equiv \partial_{\phi_1}A$, $B' \equiv \partial_{\theta_1}B$). The symbols L_1 and L_2 denote the left fixed points of the transfer matrices associated with the (C, D) and (A, B) layers, respectively.

By contracting the relevant two-layer tensor networks with one insertion of a tangent tensor on the bra/ket legs, the diagonal entries are

$G_{\phi_1,\phi_1} =$ 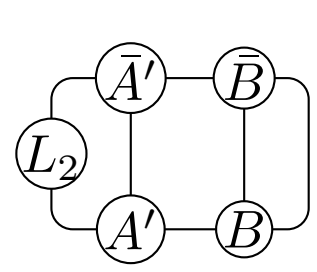

$= \dfrac{1+\cos(2\theta_1)\cos(2\theta_2)}{2}$, $G_{\theta_1,\theta_1} =$ 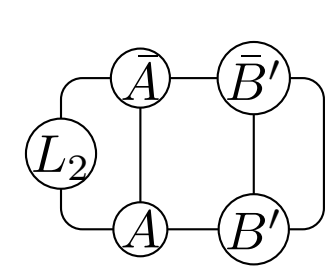

$= 1,$ (S16)

$G_{\phi_2,\phi_2} =$ 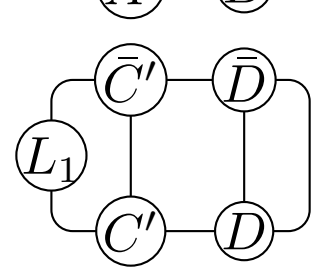

$= \dfrac{1+\cos(2\theta_1)\cos(2\theta_2)}{2}$, $G_{\theta_2,\theta_2} =$ 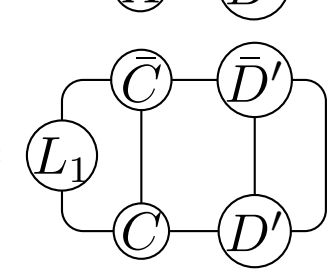

$= 1.$ (S17)

All off-diagonal elements vanish, $G_{\mu\nu} = 0$ for $\mu \neq \nu$.

B. Hamiltonian gradients

In this subsection, we calculate the explicit form of the Hamiltonian gradients part of the TDVP equation of motion:

$$h_\mu \equiv \langle \partial_{z_\mu}\psi|\hat{H}|\psi\rangle, \qquad z_\mu \in \{\theta_1,\theta_2,\phi_1,\phi_2\}. \tag{S18}$$

The Hamiltonian can be split into an SSH part $\hat{H}_0$ and a longer–range correction $\hat{V}$,

$$\begin{aligned} \hat{H} &= \hat{H}_0 + \hat{V}, \\ \hat{H}_0 &= -\sum_j \left(J_o\, \hat{\sigma}^+_{2j-1}\hat{\sigma}^-_{2j} + J_e\, \hat{\sigma}^+_{2j}\hat{\sigma}^-_{2j+1} + \text{h.c.}\right), \\ \hat{V} &= -J_3 \sum_j \left(\hat{\sigma}^+_{2j-1}\hat{\sigma}^-_{2j+2} + \text{h.c.}\right). \end{aligned} \tag{S19}$$

For clarity, we separate the contributions of $\hat{H}_0$ and $\hat{V}$ and use tensor-network diagrams to indicate the corresponding local operator insertions. We denote by h_o, h_e, and h_3 the local MPO tensors representing the odd-rung, even-bond, and next-nearest interactions, respectively; L_1 and L_2 are the left fixed points of the transfer matrices for the (C, D) and (A, B) layers.

a. SSH contribution. The three nonvanishing gradients from $\hat{H}_0$ are obtained by inserting h_o or h_e in the appropriate two-layer contractions. In diagrammatic form:

$-i\langle \partial_{\phi_1}\psi|\hat{H}_0|\psi\rangle =$ 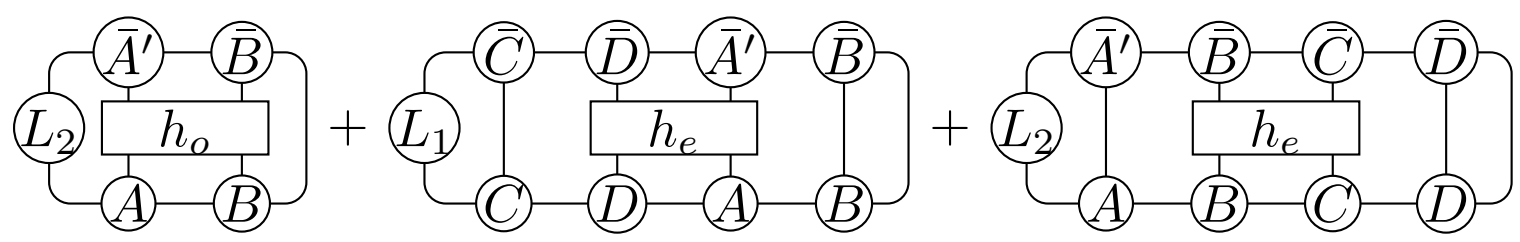

$$= \frac{J_o}{2}\left[1+\cos(2\theta_1)\cos(2\theta_2)\right] + \frac{J_e}{2}\sin(2\theta_1+2\theta_2)\sin(\phi_1)\cos(\phi_2),$$

$$-i\langle \partial_{\phi_2}\psi|\hat{H}_0|\psi\rangle = i\langle \partial_{\phi_1}\psi|\hat{H}_0|\psi\rangle\Big|^{\theta_1\leftrightarrow\theta_2}_{\phi_1\leftrightarrow\phi_2} = \frac{J_o}{2}\left[1+\cos(2\theta_1)\cos(2\theta_2)\right] + \frac{J_e}{2}\sin(2\theta_1+2\theta_2)\sin(\phi_2)\cos(\phi_1),$$

$-i\langle \partial_{\theta_1}\psi|\hat{H}_0|\psi\rangle =$ 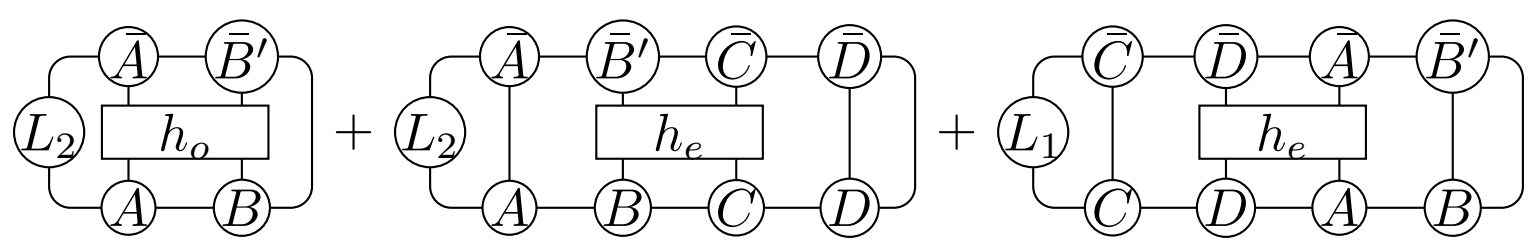 $= J_e\cos(\phi_1)\cos(\phi_2),$

$$-i\langle \partial_{\theta_2}\psi|\hat{H}_0|\psi\rangle = -\,i\langle \partial_{\theta_1}\psi|\hat{H}_0|\psi\rangle\Big|^{\theta_1\leftrightarrow\theta_2}_{\phi_1\leftrightarrow\phi_2} = J_e\cos(\phi_1)\cos(\phi_2).$$

b. Interaction contribution. The next-nearest interaction is encoded by two insertions of h_3 bridged across a layer. The corresponding gradients are

$-i\langle \partial_{\phi_1}\psi|\hat{V}|\psi\rangle =$ 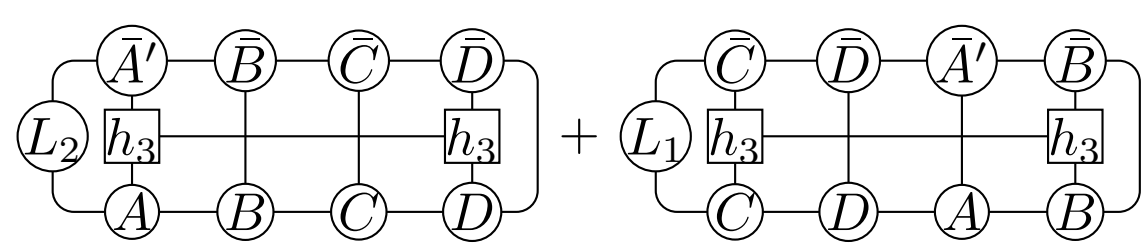 $= \frac{J_3}{2}\sin(2\theta_1+2\theta_2)\sin(\phi_2)\cos(\phi_1),$

$$-i\langle \partial_{\phi_2}\psi|\hat{V}|\psi\rangle = -\,i\langle \partial_{\phi_1}\psi|\hat{V}|\psi\rangle\Big|^{\theta_1\leftrightarrow\theta_2}_{\phi_1\leftrightarrow\phi_2} = \frac{J_3}{2}\sin(2\theta_1+2\theta_2)\sin(\phi_1)\cos(\phi_2),$$

$-i\langle \partial_{\theta_1}\psi|\hat{V}|\psi\rangle =$ 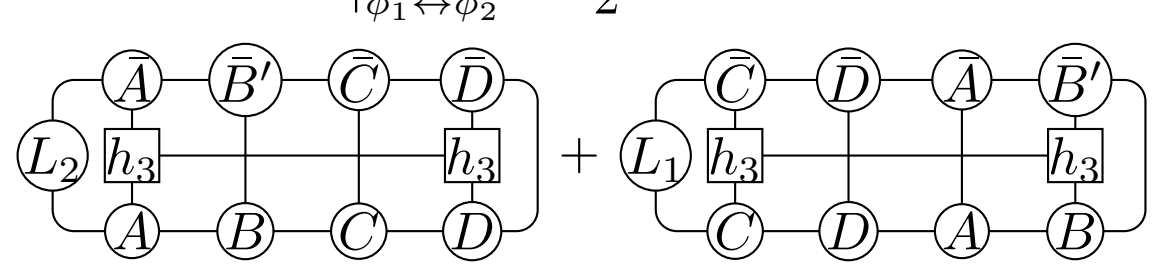 $= -J_3\cos^2(2\theta_2)\sin(\phi_1)\sin(\phi_2),$

$$-i\langle \partial_{\theta_2}\psi|\hat{V}|\psi\rangle = i\langle \partial_{\theta_1}\psi|\hat{V}|\psi\rangle\Big|^{\theta_1\leftrightarrow\theta_2}_{\phi_1\leftrightarrow\phi_2} = -J_3\cos^2(2\theta_1)\sin(\phi_1)\sin(\phi_2).$$

Collecting the SSH and interaction pieces, the Hamiltonian gradients entering the TDVP read

$$-\,i\langle \partial_{\theta_1}\psi \mid \hat{H} \mid \psi\rangle = J_e\,\cos\phi_1\cos\phi_2 - J_3\,\cos^2(2\theta_2)\,\sin\phi_1\sin\phi_2, \tag{S20}$$

$$-\,i\langle \partial_{\theta_2}\psi \mid \hat{H} \mid \psi\rangle = J_e\,\cos\phi_1\cos\phi_2 - J_3\,\cos^2(2\theta_1)\,\sin\phi_1\sin\phi_2, \tag{S21}$$

$$-\,i\langle \partial_{\phi_1}\psi \mid \hat{H} \mid \psi\rangle = \frac{J_o}{2}[1+\cos(2\theta_1)\cos(2\theta_2)] + \frac{\sin(2\theta_1+2\theta_2)}{2}\left(J_e\,\sin\phi_1\cos\phi_2 + J_3\,\sin\phi_2\cos\phi_1\right), \tag{S22}$$

$$-\,i\langle \partial_{\phi_2}\psi \mid \hat{H} \mid \psi\rangle = \frac{J_o}{2}[1+\cos(2\theta_1)\cos(2\theta_2)] + \frac{\sin(2\theta_1+2\theta_2)}{2}\left(J_e\,\sin\phi_2\cos\phi_1 + J_3\,\sin\phi_1\cos\phi_2\right). \tag{S23}$$

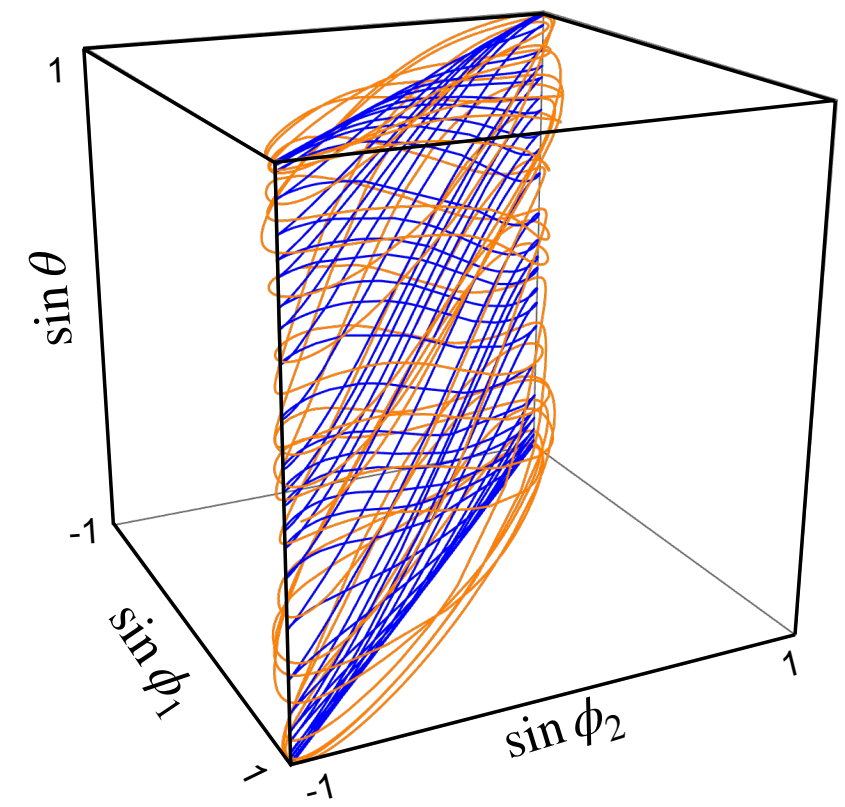

Figure S3. **Impact of flow-invariant planes.** Comparison between a TDVP trajectory constrained exactly to the invariant plane [initial condition $(\theta, \phi_1, \phi_2) = (0, 0, 0)$, blue] and one initialized slightly away from it [$(\theta, \phi_1, \phi_2) = (0, 0, 0.05\pi)$, orange]. On the invariant plane $\phi_1 = \phi_2$, the TDVP equations reduce to two variables and produce strictly regular closed orbits, whereas trajectories launched near the plane initially follow the regular orbit but gradually drift into the surrounding phase space.

C. Equations of motion

With the explicit form of the Gram matrix and Hamiltonian gradients, we obtain the TDVP equations of motion:

$$\begin{aligned}
\dot{\theta}_1 &= J_e \cos(\phi_1)\cos(\phi_2) - J_3 \cos^2(2\theta_2)\sin(\phi_1)\sin(\phi_2),\\
\dot{\theta}_2 &= J_e \cos(\phi_1)\cos(\phi_2) - J_3 \cos^2(2\theta_1)\sin(\phi_1)\sin(\phi_2),\\
\dot{\phi}_1 &= J_o + \frac{\sin(2\theta_1 + 2\theta_2)\left[J_e \sin(\phi_1)\cos(\phi_2) + J_3 \sin(\phi_2)\cos(\phi_1)\right]}{1 + \cos(2\theta_1)\cos(2\theta_2)},\\
\dot{\phi}_2 &= J_o + \frac{\sin(2\theta_1 + 2\theta_2)\left[J_e \sin(\phi_2)\cos(\phi_1) + J_3 \sin(\phi_1)\cos(\phi_2)\right]}{1 + \cos(2\theta_1)\cos(2\theta_2)}.
\end{aligned} \tag{S24}$$

It is advantageous to reorganize the dynamics in terms of symmetric and antisymmetric combinations of the variational parameters,

$$\theta_{S/A} = \tfrac{1}{2}(\theta_1 \pm \theta_2), \qquad \phi_{S/A} = \tfrac{1}{2}(\phi_1 \pm \phi_2). \tag{S25}$$

In this basis, the equations of motion acquire a more compact and transparent form,

$$\begin{aligned}
\dot{\theta}_S &= J_e\big[\cos^2(\phi_S) - \sin^2(\phi_A)\big] - \tfrac{J_3}{2}\Big[\cos(4\theta_A)\cos(4\theta_S) + 1\Big]\Big[\sin^2(\phi_S) - \sin^2(\phi_A)\Big],\\
\dot{\theta}_A &= \tfrac{J_3}{4}\,\sin(4\theta_A)\sin(4\theta_S)\Big[\cos(2\phi_S) - \cos(2\phi_A)\Big],\\
\dot{\phi}_S &= J_o + \frac{(J_e + J_3)\,\sin(4\theta_S)\,\sin(2\phi_S)}{2 + \cos(4\theta_S) + \cos(4\theta_A)},\\
\dot{\phi}_A &= \frac{(J_e - J_3)\,\sin(4\theta_S)\,\sin(2\phi_A)}{2 + \cos(4\theta_S) + \cos(4\theta_A)}.
\end{aligned} \tag{S26}$$

Several consistent reductions of this system are possible depending on the choice of initial conditions. First, if the system is initialized symmetrically with $\theta_1 = \theta_2$, as we do in the main text, then $\theta_A \equiv 0$ is preserved by the dynamics, reducing the effective dimensionality to three variables (θ, ϕ_S, ϕ_A) with $\theta \equiv \theta_S$. The equations of motion then simplify to

$$\begin{aligned}
\dot{\theta} &= J_e\big[\cos^2(\phi_S) - \sin^2(\phi_A)\big] - J_3\cos^2(2\theta)\Big[\sin^2(\phi_S) - \sin^2(\phi_A)\Big],\\
\dot{\phi}_S &= J_o + \frac{(J_e + J_3)\,\sin(4\theta)\,\sin(2\phi_S)}{3 + \cos(4\theta)},\\
\dot{\phi}_A &= \frac{(J_e - J_3)\,\sin(4\theta)\,\sin(2\phi_A)}{3 + \cos(4\theta)}.
\end{aligned} \tag{S27}$$

We integrate these equations with the adaptive Runge-Kutta solver `Tsit5` from Julia's `OrdinaryDiffEq` package, using relative tolerance 10^{-10} and absolute tolerance 10^{-12}. The Poincaré section is built by recording (θ, ϕ_2) whenever a trajectory crosses $\phi_1 = 0 \bmod 2\pi$, with the crossing point obtained by linear interpolation between the last sample before and the first sample after the crossing. This last step is important in practice: without it, the section visibly thickens at finite integrator steps. The resulting section points appear as the black dots overlaid in the main text figures, Fig. 1**c**, **d**.

A further reduction occurs when the phases are initially aligned, $\phi_1 = \phi_2 \equiv \phi$, which enforces $\phi_A \equiv 0$. In this case the system closes on the two-dimensional manifold (θ, ϕ),

$$\begin{aligned} \dot{\theta} &= J_e \cos^2 \phi - J_3 \cos^2(2\theta)\, \sin^2 \phi, \\ \dot{\phi} &= J_o + \frac{(J_e + J_3)\, \sin(4\theta)\, \sin(2\phi)}{3 + \cos(4\theta)}. \end{aligned} \tag{S28}$$

This final reduction is particularly important: as a two-dimensional dynamical system it cannot exhibit chaos, which explains the absence of irregular trajectories along the corresponding boundary of phase space. Fig. S3 illustrates this behavior: trajectories initialized exactly on the invariant plane remain confined to smooth, closed orbits, while those launched slightly away from it initially shadow the regular orbit before slowly drifting through the surrounding phase space, though they remain close to the invariant surface.

D. Exact rainbow scar point

When $J_3 = -J_e$, the model contains an exact solution associated with so-called rainbow scars [2, 3]. At this exact point, our ansatz faithfully capture the scar dynamics, where the TDVP equations of motion become:

$$\begin{aligned} \dot{\theta}_1 &= J_e \left[\cos(\phi_1)\cos(\phi_2) + \cos^2(2\theta_2)\sin(\phi_1)\sin(\phi_2)\right], \\ \dot{\theta}_2 &= J_e \left[\cos(\phi_1)\cos(\phi_2) + \cos^2(2\theta_1)\sin(\phi_1)\sin(\phi_2)\right], \\ \dot{\phi}_1 &= J_o + J_e \frac{\sin(2\theta_1 + 2\theta_2)\sin(\phi_1 - \phi_2)}{1 + \cos(2\theta_1)\cos(2\theta_2)}, \\ \dot{\phi}_2 &= J_o - J_e \frac{\sin(2\theta_1 + 2\theta_2)\sin(\phi_1 - \phi_2)}{1 + \cos(2\theta_1)\cos(2\theta_2)}. \end{aligned} \tag{S29}$$

The scar initial state corresponds to the initialization $(\theta_1, \theta_2, \phi_1, \phi_2) = (0, 0, 0, \pi/2)$. In this case, the dynamics reduce further to

$$\begin{aligned} \dot{\theta}_1 &= \dot{\theta}_2 = 0, \\ \dot{\phi}_1 &= \dot{\phi}_2 = J_o, \end{aligned} \tag{S30}$$

which is exactly the rainbow scar dynamics [3].

S5. POINCARÉ SECTIONS FOR DIFFERENT COUPLINGS AND UNDER PERTURBATIONS

In this section, we study the evolution of the Poincaré sections for different choices of parameters and boundary conditions, as well as an experimentally-relevant perturbation.

First, we analyze the Poincaré section on the plane $\phi_1 = 0$, starting from initial conditions $\theta_1 = \theta_2 = \theta, \phi_1 = 0$. The resulting Poincaré sections are shown in Fig. S4. In Fig. S4**a-d**, we show the noninteracting case where $J_3 = 0$; in Fig. S4**e-f**, we fix $J_e = -J_3$; in Fig. S4**g-h**, we choose generic couplings. In all three cases, we observe both mixed-phase and regular patterns in the phase space. It is important to note that the details of the phase space profile are sensitive to the difference in the strength of J_e and J_3. When $|J_e - J_3| < 0.5$, the profile is simpler and consists of a couple of large islands, while otherwise these islands develop substructure and smaller tori also appear outside of these islands.

Next, we analyze the effect of boundary conditions. We perform exact diagonalization of a 16-qubit chain under both open and periodic boundary conditions. As shown in Fig. S5**a**,**b**, the qualitative structure of the revival map is nearly identical in the two cases, indicating that the coexistence of regular and chaotic regions is not an artifact of boundary condition.

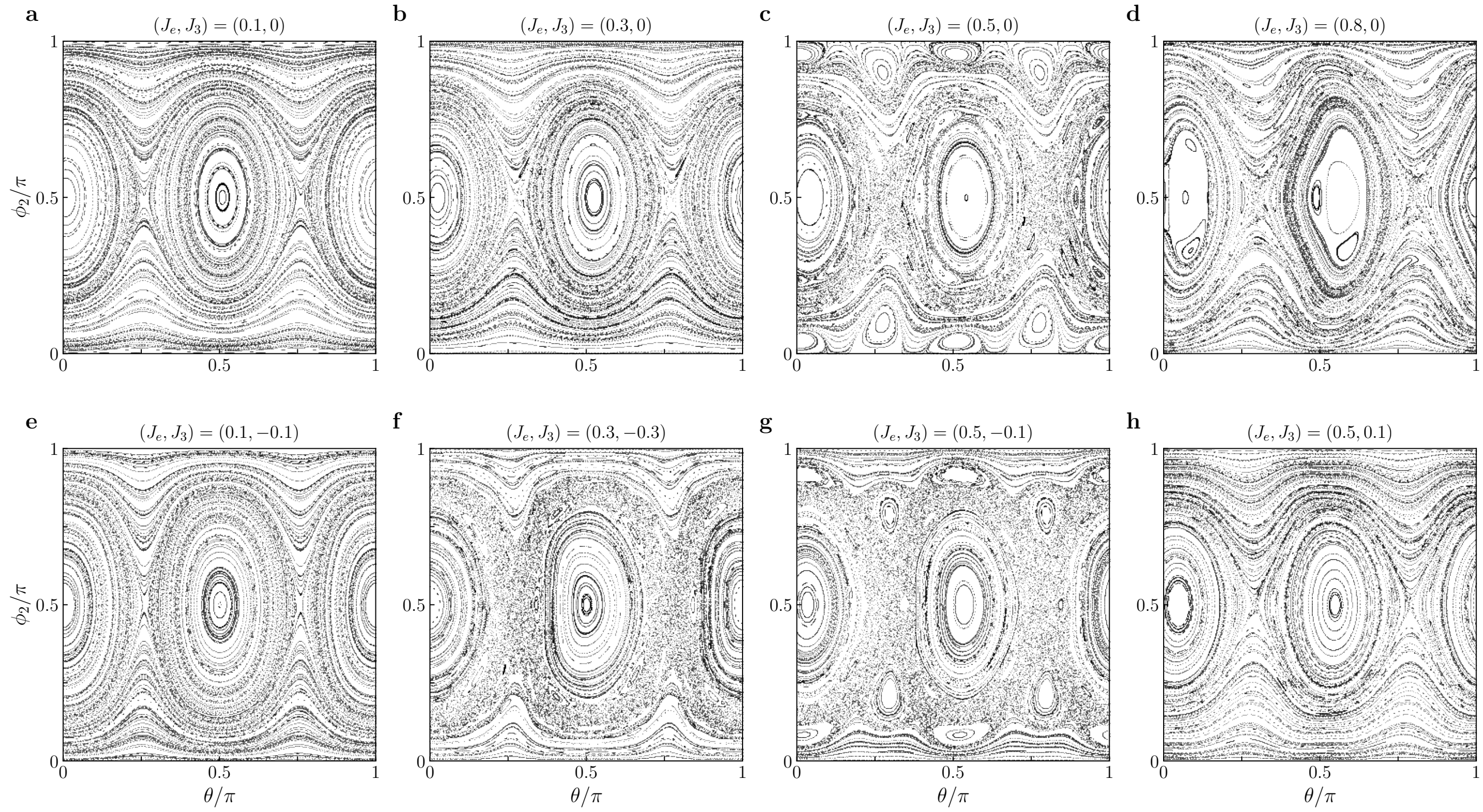

Figure S4. **Evolution of the Poincaré section upon varying the circuit parameters.** Poincaré sections on the plane $\phi_1 = 0$, starting from the MPS ansatz with parameters $\theta_1 = \theta_2 = \theta, \phi_1 = 0$. We fix $J_o = 1.0$, and the other two couplings are indicated in the title of each plot.

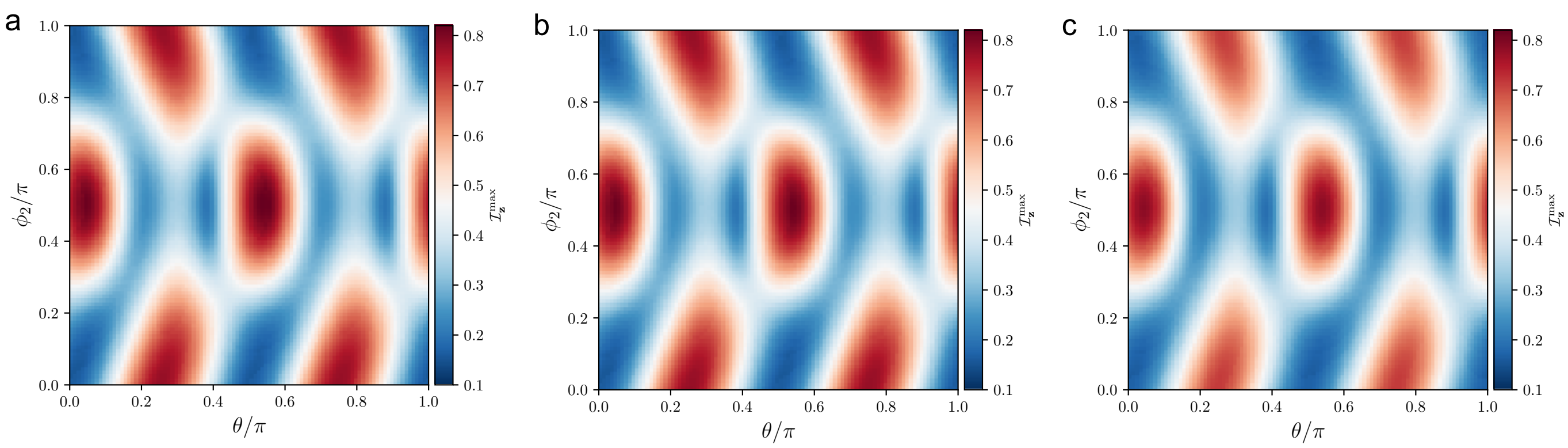

Figure S5. **Insensitivity of the Poincaré section to perturbations and type of boundary conditions. a,b** First revival peak in local observables from exact diagonalization, for the Hamiltonian under **a** open boundary conditions and **b** periodic boundary conditions. **c** First revival peak for the Hamiltonian with diagonal perturbation in Eq. (S31) with $J_d = 0.1 J_o$. All simulations are performed using exact diagonalization of a 16-qubit system.

Finally, we explore the effect of an experimentally-relevant diagonal perturbation:

$$\hat{V}_d = -J_d \sum_j \left(\hat{\sigma}_j^+ \hat{\sigma}_{j+2}^- + \hat{\sigma}_j^- \hat{\sigma}_{j+2}^+ \right), \tag{S31}$$

which introduces weak next-nearest-neighbor exchange along the ladder. In superconducting-qubit implementations, such diagonal couplings naturally arise from residual capacitive or inductive crosstalk between nonadjacent qubits, or from higher-order virtual processes mediated by the tunable couplers. These mechanisms effectively generate weak flip-flop interactions between qubits on opposite legs of the ladder, corresponding to the diagonal links in the model. We fix $J_d = 0.1 J_o$—the scale of perturbation typically observed experimentally—and compute the first revival peak of the local observable. As shown in Fig. S5**c**, the resulting phase-space pattern remains qualitatively unchanged,

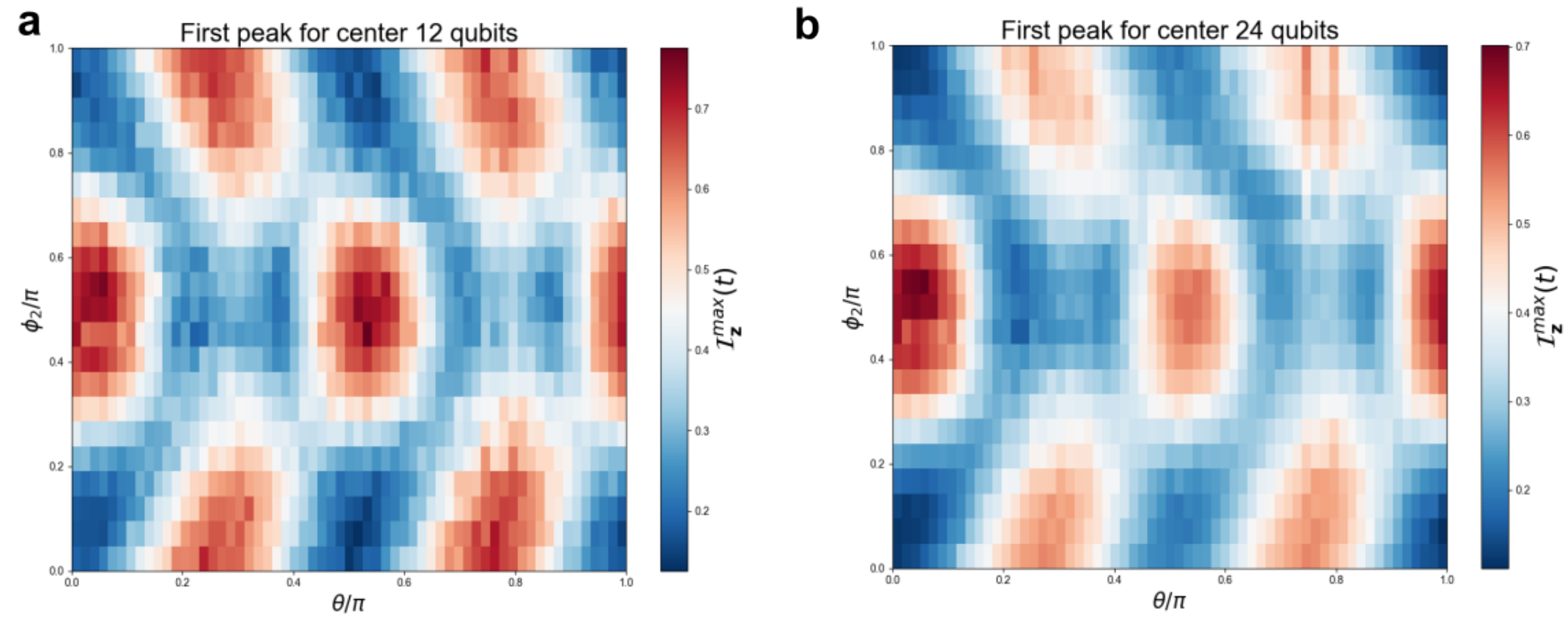

Figure S6. **Experimental observation of the first revival peak via imbalance in the 24-qubit system**. To demonstrate the robustness of mixed phase space, we contrast the imbalance measured on (a) the central 12 qubits, and (b) all 24 qubits of the ladder. The larger fluctuations in panel (b) are attributed to the boundary effects. The coupling parameters are $J_o/2\pi = 5$ MHz, $J_e/2\pi = 3$ MHz, and $J_3/2\pi = 0.25$ MHz.

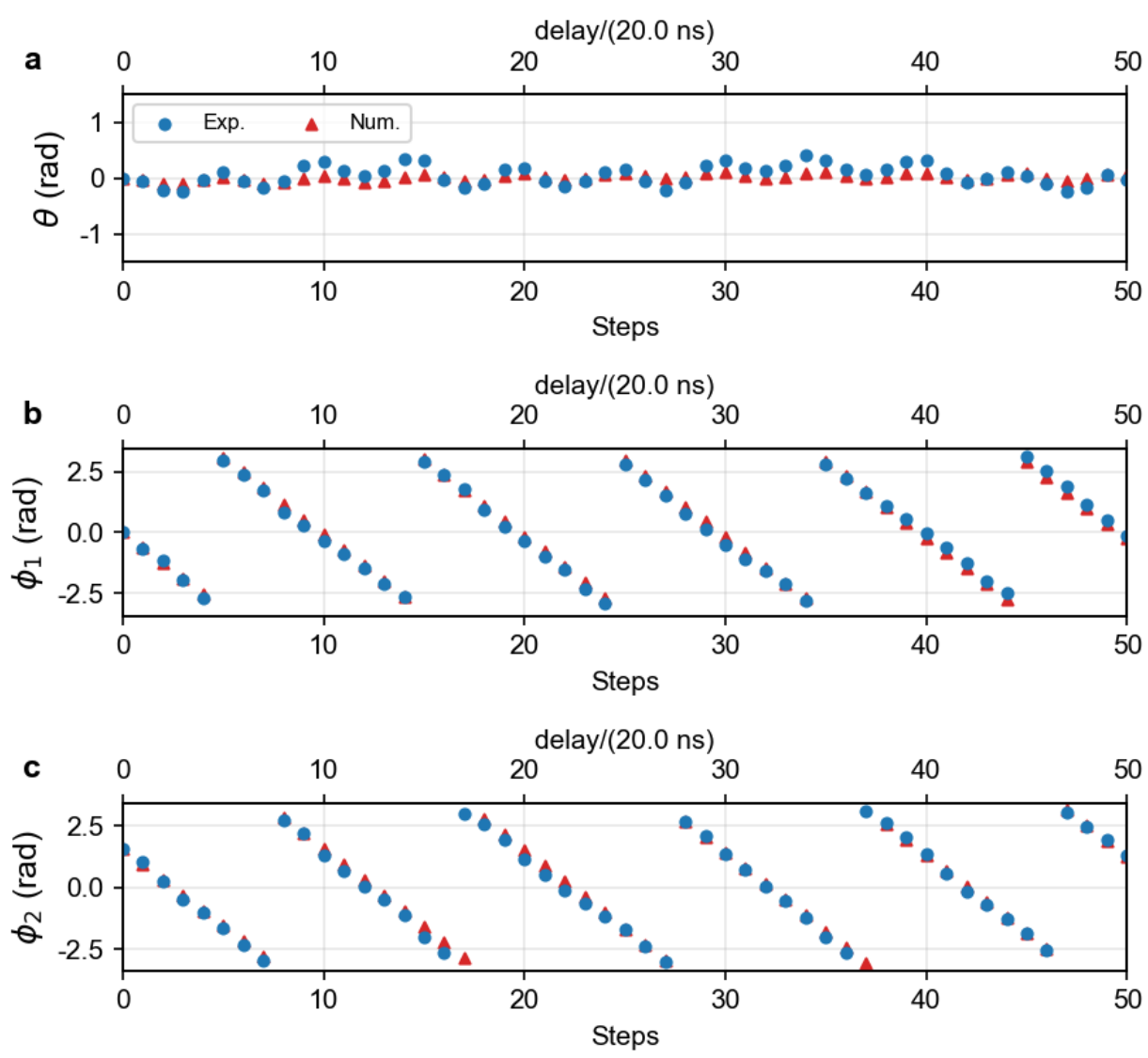

Figure S7. **The dynamics of the hybrid feedback dynamics compared to the numerical results** (a–c) Evolution of the parameters (θ, ϕ_1, ϕ_2) under the hybrid feedback protocol, starting from the matrix product state $(0, 0, 0.5\pi)$ with a fixed delay of 20 ns. The numerical results are shown for comparison. The remarkable agreement between both methods—even after a large number of steps—demonstrates the effectiveness of the hybrid feedback approach in capturing the essential dynamics of the system.

confirming that the mixed-phase structure is robust against this types of hardware imperfections. This resilience highlights that the observed coexistence of regular and chaotic trajectories is an intrinsic feature of the variational dynamics, rather than a fine-tuned property of the idealized Hamiltonian.

The robustness of the mixed phase-space structure is further illustrated in Fig. S6, which compares the imbalance-based Poincaré sections obtained from the central 12-qubit block with those extracted from all 24 qubits. While the full-system measurement reproduces the same qualitative features, including the location of the regular islands and the surrounding chaotic regions, the boundary spins introduce additional fluctuations. For this reason, the central subsystem provides a cleaner and more reliable experimental probe of the underlying phase-space structure, and is used throughout the main text.

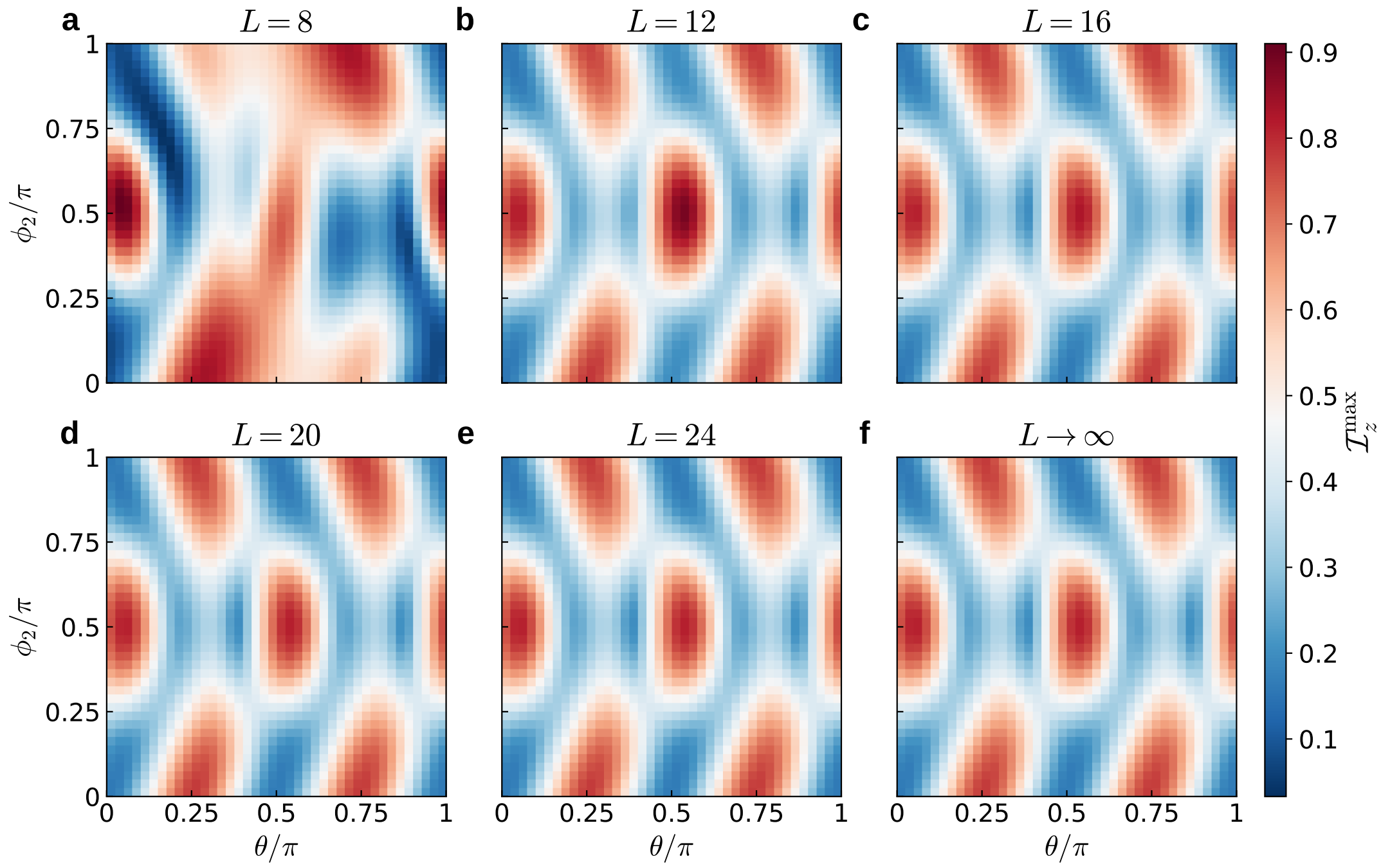

Figure S8. **Finite-size convergence of the Poincaré section.** First revival peak of the rotated imbalance $\mathcal{I}_{\boldsymbol{z}}^{\max}$ mapped over the variational parameter space (θ, ϕ_2) for system sizes $L = 8$ (**a**), 12 (**b**), 16 (**c**), 20 (**d**), 24 (**e**), and $L \to \infty$ (**f**). Panels **a**–**e** are obtained from TEBD simulations with open boundary conditions (bond dimension $\chi = 64$, time step $\delta t = 0.05\, J_o^{-1}$, total evolution time $T = 10\, J_o^{-1}$), while panel **f** is computed via iTEBD in the thermodynamic limit. All panels share the same color scale. The qualitative features, with regular islands (warm colors) and chaotic sea (cold colors), are robust across all system sizes with $L \gtrsim 12$, confirming that the observed mixed phase-space structure in the main text is not a finite-size artifact.

S6. FINITE-SIZE SCALING OF THE POINCARÉ SECTION

To verify that the mixed phase-space structure is not an artifact of finite system size, we compute the first-revival-peak maps $\mathcal{I}_{\boldsymbol{z}}^{\max}(\theta, \phi_2)$ for system sizes $L = 8 - 24$ with open boundary conditions and compare them against the thermodynamic limit $L \to \infty$. For finite systems, we employ TEBD [4] with a second-order Suzuki–Trotter decomposition (time step $\delta t = 0.05\, J_o^{-1}$, maximum bond dimension $\chi = 64$), using the same coupling parameters (J_o, J_e, J_3) as in the main text. The rotated imbalance is measured on a central 4-site block to minimize boundary effects. For the thermodynamic limit, we use iTEBD on an infinite MPS with a 4-site unit cell. The results are shown in Fig. S8. The regular island and chaotic sea are already recognizable at $L = 12$. As L increases, the contrast sharpens and the island becomes better defined. By $L = 16$ (panel **c**), the map is quantitatively indistinguishable from $L = 20$ and $L = 24$ (panels **d**, **e**), and matches the $L \to \infty$ result (panel **f**) closely.

The rapid convergence with L has a simple physical origin. The first revival occurs on a time scale set by the local coupling J_o, which is small enough that correlations have not propagated to the boundaries for $L \gtrsim 16$, consistent with Lieb–Robinson bounds [5]. The imbalance measured on the central subsystem therefore probes local dynamics and is insensitive to the system edges. These results confirm that the coexistence of regular and chaotic trajectories is an intrinsic property of the SSH ladder. In the main text, ED calculations in Fig. 2**c** were performed at $L = 16$ which yields a converged Poincaré map (for convenience, we typically use periodic boundary conditions for ED simulations, however the finite-size corrections to the first revival peak are negligible at this system size).

S7. REALISTIC MODELING OF THE EXPERIMENT

The coherence times of the device used in the main text are much longer than the observation time window: compare the median values $T_1 = 73.6\,\mu$s and $T_2 = 17.4\,\mu$s with the total observation window of only $\sim 320\,$ns. On the latter timescale, dephasing and relaxation only weakly perturb the SSH dynamics and do not change the qualitative distinction between regular and irregular trajectories. This is confirmed by looking directly at the imbalance operator.

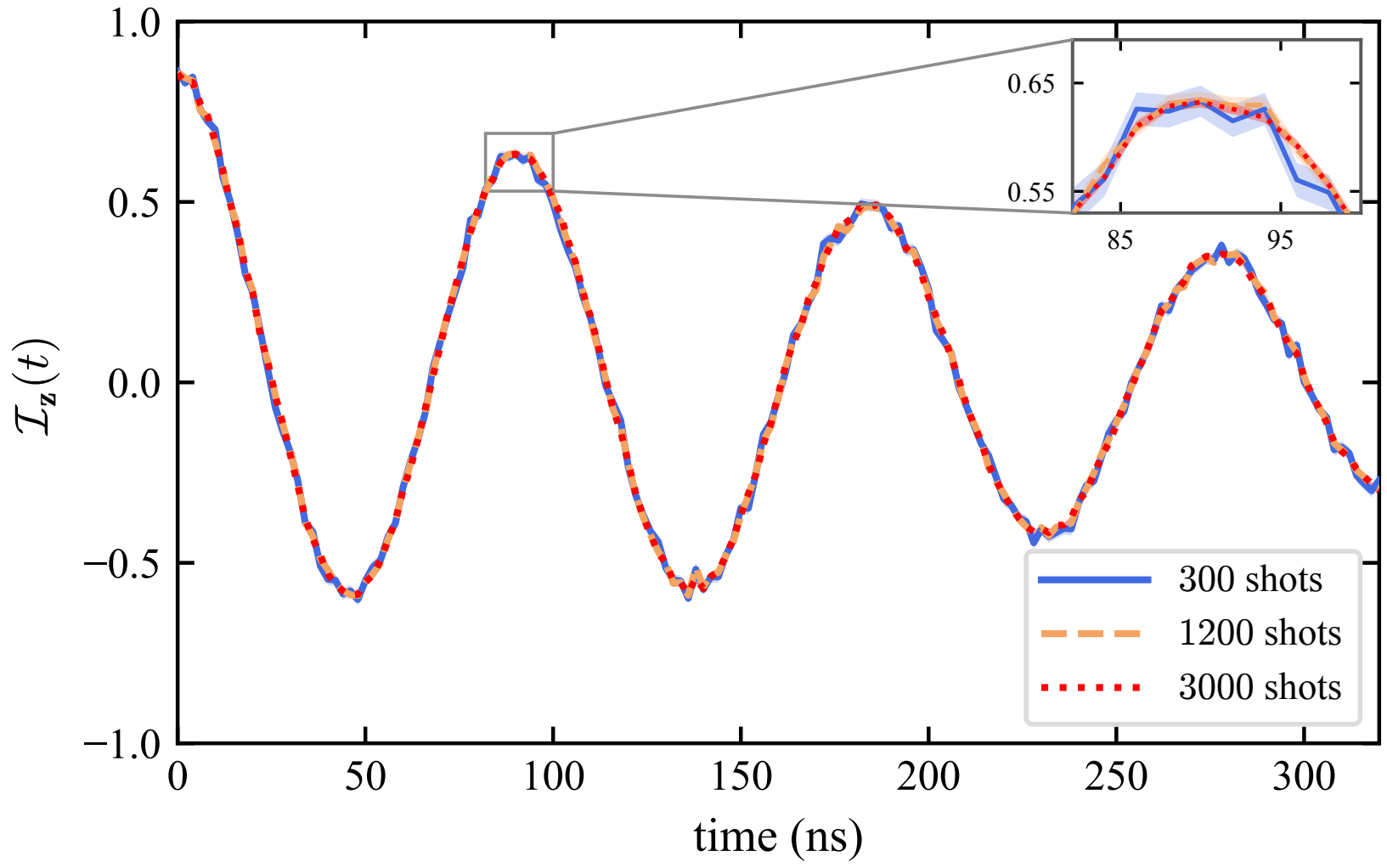

Figure S9. **Shot-count convergence of the imbalance dynamics.** Imbalance dynamics obtained using 300, 1200, and 3000 shots per data point. Shaded bands denote the 1σ statistical uncertainty of the mean, estimated from single-shot fluctuations. The curves overlap almost completely over the full time trace. Inset: At 300 shots, the relative statistical error is only 1.1%, while increasing to 3000 shots reduces it to 0.4%.

Before the outer pair rotations, the central two-site piece is $o_2 = |01\rangle\langle 01| - |10\rangle\langle 10|$, and the inner-bond rotation gives

$$U_{xy}(\theta)\, o_2\, U_{xy}(-\theta) = \cos(2\theta)\, o_2 + i\sin(2\theta)\big(|01\rangle\langle 10| - |10\rangle\langle 01|\big).$$

The dephasing-sensitive part is therefore the single coherence $|01\rangle\langle 10|$, whose two basis states differ on two qubits and hence decay at the combined rate $2/T_2$. This gives the conservative envelope $|\delta\mathcal{I}_z(t)| \lesssim 1 - e^{-2t/T_2} = 2t/T_2 + O((2t/T_2)^2)$. Over the full ~ 320 ns window this yields $1-e^{-2t/T_2} \approx 3.6\%$, while the truncation error of the linearized estimate is only $(2t/T_2)^2/2 \approx 6.8\times10^{-4}$. Since the full four-site imbalance is just a unitary dressing of this normalized local operator, this sets the natural error-scale estimate.

The device-rate Lindblad simulations are consistent with the estimate above. Using single-site amplitude damping and pure dephasing at the median device rates on an $L = 24$ open chain, the mean root-mean-square deviation across the three Fig. 2**b** imbalance cuts is only $\sim 2.5\times10^{-3}$, and the largest pointwise deviation stays below 8.5×10^{-3}. For the θ value shown in Fig. S10, the maximum difference is on the order $\sim 10^{-3}$, hence we magnify it in the inset. The subsystem-fidelity traces exhibit similarly small deviations (data not shown).

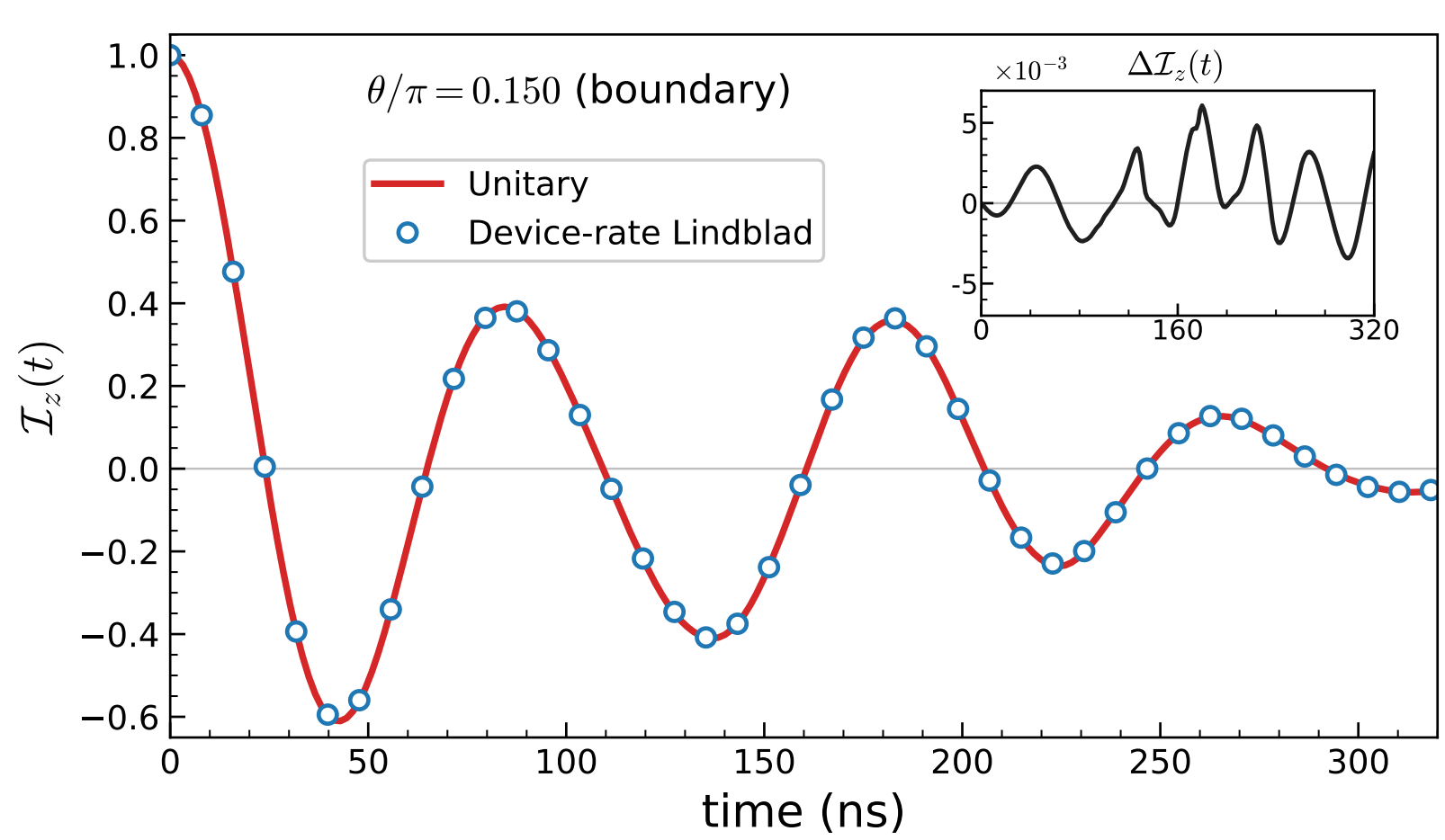

Figure S10. **Imbalance under realistic decoherence rate.** Red line: unitary evolution at the boundary value $\theta/\pi = 0.150$ between regular and chaotic dynamics. Blue open circles: Lindblad result using T_1 and T_2 values measured on the device (see text). Inset: the difference between unitary and Lindblad dynamics, $\Delta\mathcal{I}_z(t)$, reveals a small difference on the order $\sim 10^{-3}$ over the full ~ 320 ns window. Data obtained using TEBD without the weak diagonal coupling $J_d = 0.1J_o$.

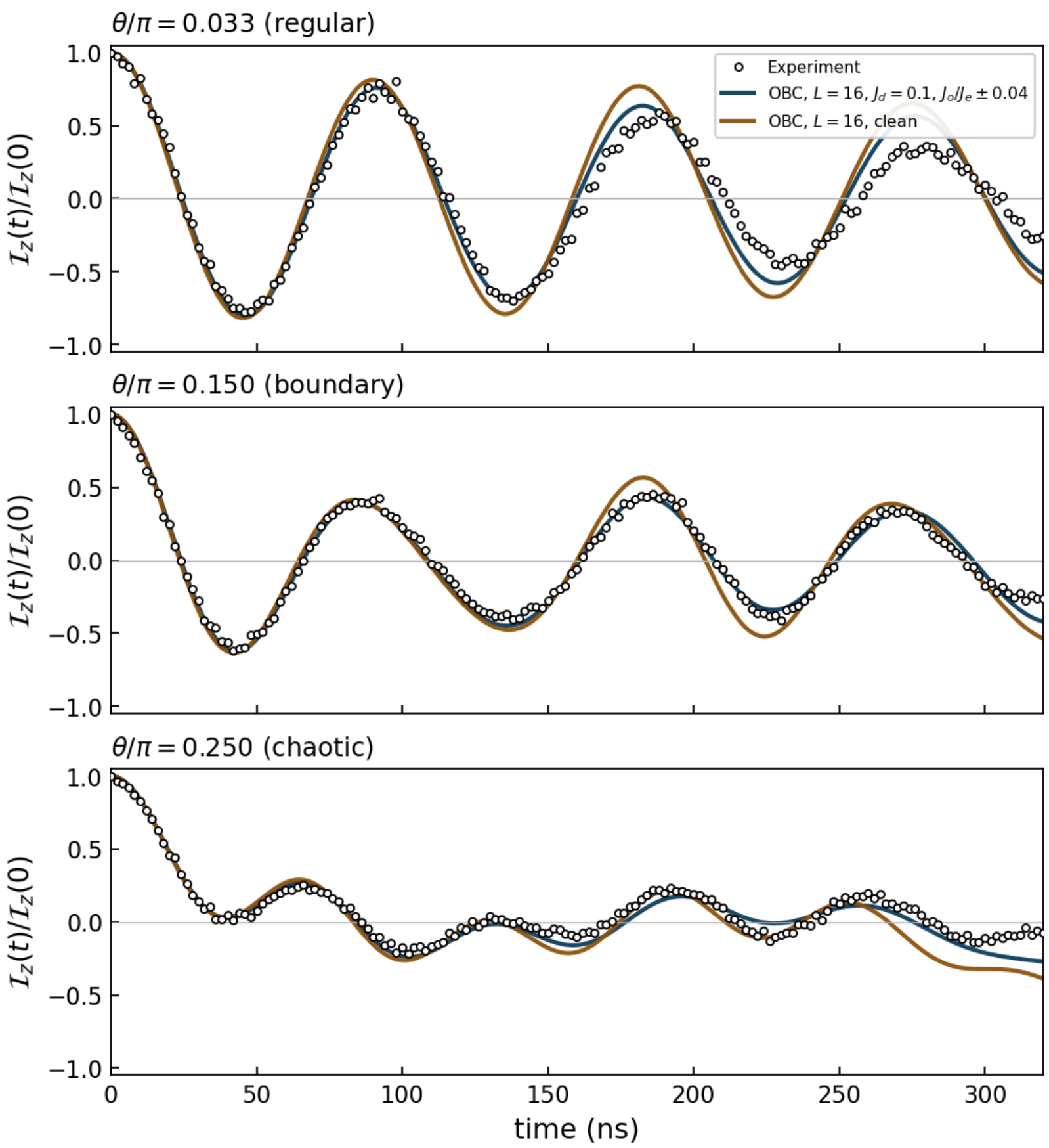

Figure S11. **Normalized imbalance dynamics with realistic coherent imperfections.** Experimental imbalance traces reproduced from Fig. 2**b** of the main text (black open circles), compared with unitary simulations on a 16-qubit open chain (solid lines). The numerics are either for a clean system (orange lines) or including a weak next-nearest-neighbor (diagonal) coupling $J_d = 0.1\,J_o$ and static uniform bond disorder of half-width $0.04\,J_o$ added independently to each J_o and J_e hopping (blue lines). The three panels correspond to $\theta/\pi = 0.033$ (top, regular), 0.150 (middle, regular/chaotic boundary), and 0.250 (bottom, chaotic), with $\phi_1 = 0$ and $\phi_2 = \pi/2$. To suppress the imperfect initial-state preparation, we normalize the imbalance according to Eq. (S33). Once the coherent Hamiltonian perturbations are included, the numerical traces track more closely the measured data across all three trajectories over the full time window, supporting the interpretation that the residual deviation from the clean SSH numerics originates in small coherent device imperfections rather than in decoherence or finite-size effects.

Therefore, the mismatch between the measured Fig. 2**b** traces and the clean numerics is not due to dephasing (or finite-size effects, see Sec. S6). Instead, we attribute it to two coherent imperfections that are absent from the idealized SSH Hamiltonian. The first is the weak next-nearest-neighbor (diagonal) exchange $\hat{V}_d$, already introduced in Eq. (S31). In superconducting-qubit devices, this coupling is generated by residual capacitive/inductive crosstalk and by higher-order virtual processes through the tunable couplers; we fix its amplitude at the experimentally representative value $J_d = 0.1\,J_o$. The second is the static fabrication and calibration disorder of the intra-/inter-dimer hoppings, which we model as independent uniform perturbations on each bond, $J_{o,j} = J_o + \delta J_{o,j}$, $J_{e,j} = J_e + \delta J_{e,j}$, where $\delta J_{o,j}$, $\delta J_{e,j}$ are drawn uniformly from an interval $[-w, +w]$, with $w = 0.04\,J_o$. The Hamiltonian used in the realistic simulation is then

$$\hat{H}_{\rm real} = \sum_j J_j^{\rm dis}\big(\hat{S}_j^+\hat{S}_{j+1}^- + {\rm h.c.}\big) + J_3 \sum_{j\in{\rm odd}} \big(\hat{S}_j^+\hat{S}_{j+3}^- + {\rm h.c.}\big) + J_d \sum_j \big(\hat{S}_j^+\hat{S}_{j+2}^- + {\rm h.c.}\big), \tag{S32}$$

with $J_j^{\rm dis} = J_{o,j}$ on odd (strong-dimer) bonds and $J_j^{\rm dis} = J_{e,j}$ on even (weak inter-dimer) bonds, and $J_3 = 0.05\,J_o$ as in the base model.

A further, non-dynamical contribution enters through the initial-state preparation and the reverse-rotation gates $U_M^\dagger(\boldsymbol{z})$ used to access the rotated-imbalance observable: these are realized with finite-fidelity single- and two-qubit digital gates (Fig. S2), which produces a time-independent offset on the nominal value $\mathcal{I}_z(0) = 1$ that does not reflect

the many-body evolution. While this is difficult to faithfully model, we simply remove this offset by normalizing the imbalance

$$\tilde{\mathcal{I}}_z(t) \equiv \frac{\mathcal{I}_z(t)}{\mathcal{I}_z(0)}, \tag{S33}$$

so that every experimental curve starts from unity by construction.

Before comparing the experimental traces with realistic simulations, we also verify that the shot count used for the main-text imbalance measurements is sufficient. Fig. S9 shows a representative convergence test of the imbalance dynamics using 300, 1200, and 3000 shots per data point, processed with the same readout-error mitigation used elsewhere in the experiment. Across the full time window, the three traces agree within their statistical uncertainties, indicating that 300 shots are sufficient to resolve the imbalance dynamics reported in the main text.

The simulations of the normalized imbalance, Eq. (S33), under the Hamiltonian in Eq. (S32) for a single realization of the couplings, are presented in Fig. S11 for $L = 16$ qubits with open boundary conditions. The three representative trajectories (regular, boundary, and chaotic) are in better agreement with experimental data compared to Fig. 2**b** of the main text over the full $\sim 320\,\mathrm{ns}$ window, albeit with some remaining discrepancy for the regular trajectory. In conclusion, the main source of residual experiment-theory deviations are static coherent imperfections of the couplings in $\hat{H}$ and an overall attenuation of the initial imbalance from imperfect state preparation and readout.

S8. CONVERGENCE OF THE HYBRID PROTOCOL

Step 4 of the algorithm in the main text can be viewed as maximizing the fidelity, $\max_{\boldsymbol{z}} |\langle\psi(\boldsymbol{z})|\psi(t)\rangle|^2$, i.e., the overlap of the true time-evolved state with the closest variational state on $\mathcal{M}$. This step therefore realizes a discrete projection of the quantum trajectory back onto $\mathcal{M}$, with a clear physical meaning. A generic many-body state contains both a low-entanglement component $|\psi_{\mathcal{M}}\rangle$ and a highly-entangled residual $|\psi_\perp\rangle$, such that $|\psi(t)\rangle = |\psi_{\mathcal{M}}(t)\rangle + |\psi_\perp(t)\rangle$. Under chaotic evolution, $|\psi_\perp\rangle$ rapidly accumulates entanglement, causing destructive interference and dephasing in observable quantities. In contrast, $|\psi_{\mathcal{M}}\rangle$ evolves quasi-classically, following the TDVP flow and therefore preserves coherent oscillations over long times. The hybrid feedback effectively enforces the iterative map $|\psi^{(n+1)}\rangle = \mathcal{P}_{\mathcal{M}}\,\hat{U}(\Delta t)\,|\psi^{(n)}\rangle$. Repeated application of this map suppresses the entropy-generating component $|\psi_\perp\rangle$ while amplifying the smooth, quasi-regular motion within $\mathcal{M}$. When the feedback loop converges, it identifies a self-consistent fixed point

$$e^{-i\hat{H}T}\,|\psi^*\rangle \simeq e^{i\varphi}\,|\psi^*\rangle, \tag{S34}$$

corresponding to a (quasi-)periodic trajectory on the variational manifold. This orbit represents the most stable initial state accessible within the ansatz – a trajectory that minimizes variational leakage and exhibits maximally sustained coherence under repeated evolution and projection. In this sense, the hybrid feedback loop acts as a variational distillation process: each iteration filters out unstable, rapidly thermalizing motion, leaving behind the coherent low-dimensional dynamics. The corresponding experimental ScarFinder trajectory, obtained from the same update rule, is shown in Fig. S7 for comparison.

The hybrid feedback protocol can be viewed as a discrete approximation of the continuous TDVP flow, where each feedback cycle represents a finite step of duration Δt. In the limit $\Delta t \to 0$, the projection–evolution sequence becomes equivalent to the infinitesimal TDVP evolution, and the hybrid trajectory coincides with the continuous variational dynamics. For finite Δt, however, the discrete update introduces a small systematic deviation from the exact TDVP path, which acts as an effective dissipative correction. This correction gradually steers the system toward the nearest stable periodic orbit of the TDVP manifold. Fig. S12 visualizes this process on the Poincaré section of the variational manifold. The blue points represent intersections of TDVP trajectories with the chosen plane, forming closed regular orbits. The colored spirals correspond to successive iterations of the hybrid protocol for different feedback step sizes Δt. Each iteration approximates the TDVP flow but, due to the finite-step discretization, the trajectory slowly spirals inward toward the center of the regular island. The center corresponds to a locally most stable orbit, characterized by minimal leakage from the variational manifold and maximal revival fidelity.

The rate of convergence depends sensitively on the choice of Δt. Smaller Δt provides a more accurate approximation of the continuous TDVP evolution, leading to slower but smoother convergence toward the fixed point. Larger Δt accelerates the approach but also amplifies discretization errors, causing the final orbit to deviate slightly from the ideal TDVP trajectory. Consequently, the steady-state orbit obtained after many iterations is not identical for different Δt, but rather represents a deformed version of the underlying continuous orbit. This deformation vanishes smoothly in the limit $\Delta t \to 0$, confirming that the hybrid feedback protocol continuously interpolates between a dissipative discrete map and the conservative TDVP flow.

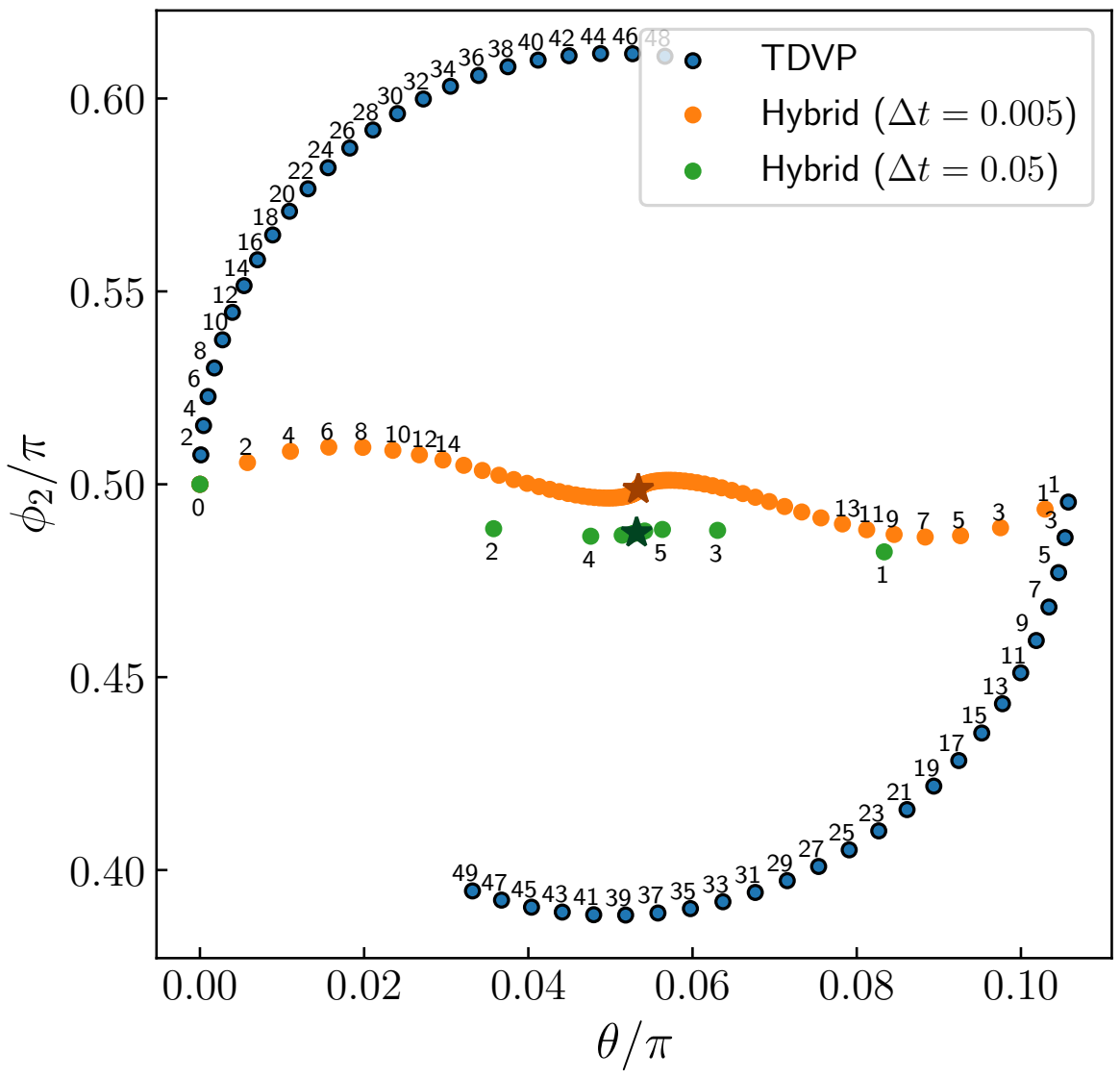

Figure S12. **Comparison between TDVP and hybrid feedback trajectories.** Poincaré section of the variational dynamics obtained from TDVP (blue points) and from the hybrid feedback algorithm (orange and green points). For small feedback step size Δt, each iteration of the hybrid protocol closely approximates continuous TDVP evolution, producing a discrete trajectory in the variational parameter space. Whenever the trajectory crosses the Poincaré plane, a point is recorded, forming the spiral patterns shown. The spiral illustrates how the iterative feedback dynamics gradually converge toward the center of the regular island, corresponding to a more stable periodic orbit. Different values of Δt lead to distinct convergence rates, and the final converged orbit is slightly deformed depending on Δt, reflecting the finite-step discretization of the continuous TDVP flow.

S9. QUANTUM MANY-BODY MIXED PHASE SPACE BEYOND LOCAL OBSERVABLES: INVERSE PARTICIPATION RATIO, ENTANGLEMENT AND PCA ENTROPY

While our study of mixed phase space in the main text focused on finite systems, here we demonstrate that the interacting SSH ladder exhibits similar ergodicity-breaking features even in the thermodynamic limit, $N\to\infty$. As explained in Sec. S4, the shallow circuit ansatz states from the main text can be conveniently expressed as infinite matrix-product states (iMPS), which we denote by $|\psi(\theta, \phi_1, \phi_2)\rangle$. Using time-evolving block decimation (TEBD) [4], we analyze the evolution of bipartite entanglement entropy, $S_E = -\mathrm{tr}\rho_A \ln \rho_A$, where ρ_A is the reduced density matrix of one half of an infinite chain. We fix $\phi_1 = 0$ and consider two representative families of initial conditions, $|\xi(\phi_2)\rangle$ belonging to the regular island and $|\eta(\phi_2)\rangle$ in the chaotic region of in Fig. 1**d** of the main text, as we vary $\phi_2 \in [0.4\pi, 0.6\pi]$. Although these states share the same energy expectation, $\langle \hat{H} \rangle = 0$, which places them at the center of the many-body spectrum, their entanglement dynamics differ qualitatively, as shown in Fig. S13**a**.

According to the ETH, states at similar energies should exhibit similar thermalization dynamics and entropy growth rates. By contrast, Fig. S13**a** reveals two clearly distinct dynamical responses: states belonging to the ξ family show slow, oscillatory growth characteristic of regular trajectories, while the η family displays rapid, almost linear entanglement growth indicative of chaotic dynamics. Similar behavior is seen in the relaxation behavior of local observables. This coexistence of distinct dynamical regimes at the same energy density is a weak violation of ETH and points to the emergence of mixed phase space structure within the variational manifold, consistent with our finite-N results in Fig. 2 of the main text.

The entanglement entropy analysis above relies on numerical simulations, since S_E is challenging to access in experiment, requiring full state tomography. To confirm the existence of mixed phase space structures beyond local observables, we next consider two observables that can be extracted directly from the experimental bitstring snapshots. The first is the principal component analysis (PCA) entropy, recently used in Refs. [6, 7] as a probe of quantum transport and ergodicity breaking. It quantifies the effective dimensionality of the measurement outcome distribution by performing a singular-value decomposition of the snapshot data matrix. At each time step t, we arrange the n_{shots} measured bitstrings as rows of a data matrix $X(t) \in \mathbb{R}^{n_{\mathrm{shots}} \times L}$ (entries 0 or 1, no centering) and compute its singular values $\{s_k\}$. The PCA entropy is the Shannon entropy of the normalized spectrum of singular values,

$$S_{\mathrm{PCA}}(t) = -\sum_k \bar{\lambda}_k \ln \bar{\lambda}_k, \tag{S35}$$

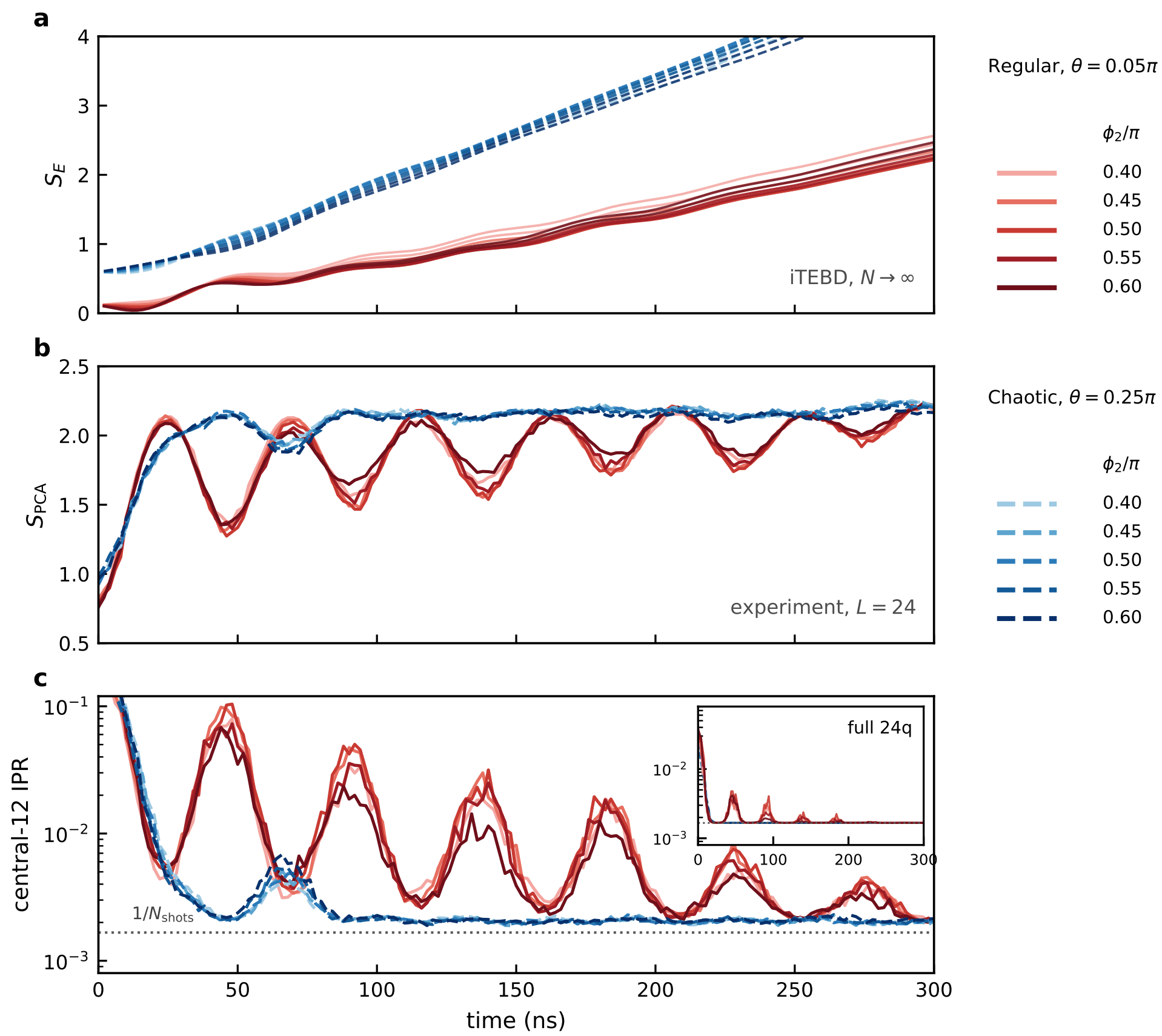

Figure S13. **Signatures of quantum many-body mixed phase space beyond local observables.** **a** Time evolution of the bipartite entanglement entropy S_E for two families of initial states within the shallow-circuit manifold. The red curves correspond to states $|\xi(\phi_2)\rangle \equiv |\psi(0.05\pi, 0, \phi_2)\rangle$ located in the regular island of phase space in Fig. 1**d** of the main text, while the blue curves correspond to chaotic states $|\eta(\phi_2)\rangle \equiv |\psi(0.32\pi, 0, \phi_2)\rangle$. The angle ϕ_2 is varied in the interval $[0.4\pi, 0.6\pi]$, as indicated by the color gradient. The distinct slopes of entropy growth reflect markedly different dynamical regimes (slow, oscillatory evolution versus rapid entanglement spreading), consistent with our Poincaré section for finite N in Fig. 2 of the main text. The simulations were performed using iTEBD on an iMPS representation with maximum bond dimension $\chi = 256$. **b** Time evolution of the PCA entropy S_{PCA}, computed directly from the experimental bitstring snapshots on the $L = 24$ qubit ladder. Red curves show initial states with $\theta = 0.05\pi$ (regular island) and blue curves show $\theta = 0.25\pi$ (chaotic sea), with $\phi_2 \in [0.4\pi, 0.6\pi]$. Regular trajectories exhibit oscillatory S_{PCA} with pronounced dips, while chaotic trajectories saturate rapidly to a near-maximal value. **c** Time evolution of the classical inverse participation ratio (IPR) for the same families of initial conditions as in **b**. The main panel shows the subsystem IPR computed from the central twelve-qubit subsystem, while the inset shows the corresponding full-chain IPR for $L = 24$ qubits as a reference. Regular trajectories display clear IPR revivals at the same characteristic times as the imbalance revivals in Fig. 2**b** of the main text, indicating periodic refocusing of the wavefunction in the computational basis. Chaotic trajectories decay rapidly to a flat baseline. We note that this baseline corresponds to the finite-sampling floor IPR $\approx 1/N_{\mathrm{shots}}$: once the state delocalizes over more basis states than the number of measured shots (here, $N_{\mathrm{shots}} \approx 600$), each shot returns a unique bitstring and the empirical IPR saturates at $1/N_{\mathrm{shots}}$ regardless of the true degree of delocalization. The contrast between regular and chaotic trajectories is nevertheless physical, as the IPR revivals of the regular family lie well above this floor.

where $\bar{\lambda}_k = s_k^2 / \sum_j s_j^2$. Ref. [6] showed that S_{PCA} serves as an experimentally accessible proxy for the entanglement entropy. In particular, in Rydberg atom arrays, S_{PCA} for scar initial states develops pronounced oscillations, distinguishing such states from thermalizing ones, where S_{PCA} rapidly approaches saturation value without persistent oscillations [7]. This makes S_{PCA} a natural substitute for S_E when only bitstring measurements are available.

Fig. S13**b** shows $S_{\mathrm{PCA}}(t)$ computed from the raw experimental snapshots on the $L = 24$ qubit ladder, for regular states at $\theta = 0.05\pi$ (red) and chaotic states at $\theta = 0.25\pi$ (blue), each at five values of $\phi_2 \in [0.4\pi, 0.6\pi]$. The two families are sharply separated: the regular family exhibits oscillatory S_{PCA} with pronounced dips, reflecting periodic refocusing of the snapshot distribution as the wave function revisits the initial manifold state; the chaotic family saturates rapidly to a near-maximal plateau, consistent with fast scrambling of the bitstring distribution.

The second observable is the inverse participation ratio (IPR), a standard measure of localization in Hilbert space:

$$\mathrm{IPR}(t) = \sum_b p(b,t)^2, \tag{S36}$$

where $p(b,t)$ is the empirical frequency of bitstring b. For a state localized on a few computational basis states, the IPR is large; for a state uniformly spread over $\mathcal{D}$ basis states, IPR $\sim 1/\mathcal{D}$. In experiment, for a fixed number of snapshots N_{shots}, the average IPR is lower-bounded by $1/N_{\text{shots}}$, hence a relatively large number of N_{shots} may be required to reveal a clear difference between regular and chaotic states on moderate timescales. Regular trajectories, where the wave function periodically refocuses, are expected to show IPR revivals, while chaotic trajectories should decay to a featureless floor $\approx 1/N_{\text{shots}}$. This is particularly clear for the IPR of the central 12-qubit subsystem in Fig. S13**c**: the regular family of initial states displays clear IPR revivals at the same characteristic times as the imbalance revivals in Fig. 2**b** of the main text, confirming periodic reconcentration in the computational basis. The chaotic family instead drops rapidly to the value $\approx 1/N_{\text{shots}}$ and remains near it, consistent with uniform spreading over the accessible Hilbert space to the extent allowed by the sampling resolution.

In summary, the entanglement entropy, PCA entropy, and IPR provide three independent types of evidence that initial states in the regular island and the chaotic sea give rise to qualitatively distinct dynamics, not only in local magnetization but also in global observables.

S10. REFINED POINCARÉ SECTION ANALYSIS

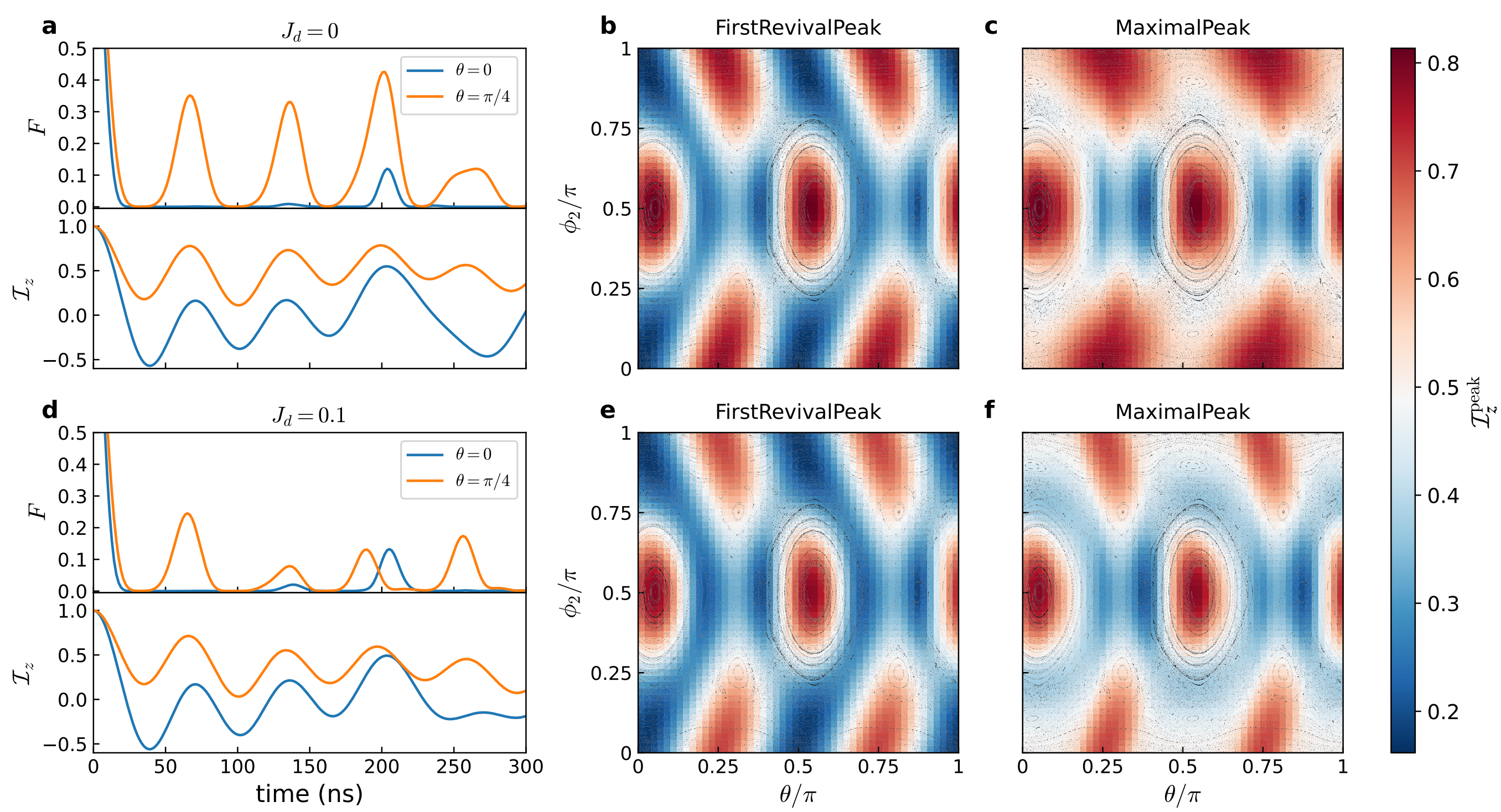

Figure S14. **Refined Poincaré section mapping.** **a**,**d** Time evolution of fidelity (top) and imbalance (bottom) for two representative initial states with $\phi_1 = \phi_2 = 0$, $\theta = 0$ (blue) and $\theta = \pi/4$ (orange), without (**a**, $J_d = 0$) and with (**d**, $J_d = 0.1$) diagonal perturbation. The two observables display qualitatively similar revival patterns. For $\theta = \pi/4$, the strongest revival occurs at the first peak, whereas for $\theta = 0$, the maximal signal appears only at the third revival. **b**,**e** First revival peak of the imbalance, $\mathcal{I}_{\boldsymbol{z}}^{\max}$, mapped as a function of (θ, ϕ_2) and overlaid with the TDVP Poincaré section (black dots). **c**,**f** Maximal revival amplitude of the imbalance, $\max_{t>t_1} \mathcal{I}_{\boldsymbol{z}}(t)$, mapped as a function of (θ, ϕ_2) and overlaid with the TDVP Poincaré section (black dots). The regions of high revival amplitude align closely with the TDVP regular islands, while the low-revival areas correspond to the chaotic sea. Compared to $\mathcal{I}_{\boldsymbol{z}}^{\max}$, this refined measurement achieves an improved agreement with the TDVP Poincaré section. The top row (**a**–**c**) shows results without diagonal perturbation ($J_d = 0$), while the bottom row (**d**–**f**) includes a nearest-neighbour perturbation $J_d = 0.1$, which partially breaks integrability and shrinks the regular islands. Numerical results are obtained by exact diagonalization of an $L = 16$ spin chain with periodic boundary conditions.

As mentioned in the main text, the Poincaré sections inferred from imbalance measurements can show small discrepancies from the TDVP predictions, e.g., near the upper and lower boundaries of the phase space in Fig. 2 of the main text. We can gain intuition about these discrepancies if we restrict to symmetric initial conditions, $\phi_1 = \phi_2 = 0$, for which the dynamics reduce to a two-dimensional submanifold. Since two-dimensional continuous dynamical systems cannot display deterministic chaos due to the Poincaré–Bendixson theorem [8], this reduction implies strictly regular, although not necessarily periodic, trajectories. Figure S14**a** illustrates this point more explicitly for fixed $\phi_1 = \phi_2 = 0$ and two states with $\theta = 0$ and $\theta = \pi/4$, which exhibit qualitatively different dynamical behavior, even though both lie within the regular region of the TDVP Poincaré section. In terms of fidelity $F(t) \equiv |\langle\psi(0)|\psi(t)\rangle|^2$ (top panel of Fig. S14**a**) the $\theta = \pi/4$ state reaches its maximal value at the first revival, whereas for $\theta = 0$ the fidelity continues to increase over several revivals, with the largest peak appearing at the third revival. Hence, a diagnostic based solely on the first peak would incorrectly classify the $\theta = 0$ dynamics as irregular, despite the clear presence of oscillations. The same trend is evident in the imbalance dynamics (bottom panel of Fig. S14**a**): for $\theta = \pi/4$, the peak amplitude of $\mathcal{I}_{\boldsymbol{z}}(t)$ decays gradually, while for $\theta = 0$, successive revivals grow in strength, mirroring the behavior observed in fidelity. Consequently, restricting the analysis to the first revival underestimates the stability of these trajectories and misrepresents the corresponding regions in the imbalance-based Poincaré map.

To overcome this limitation, we refine our diagnostic by extending the observation window in time and taking the *maximal* revival amplitude, regardless of when it occurs, $\max_{t>t_1} \mathcal{I}_{\boldsymbol{z}}(t)$, where t_1 denotes the time of the first minimum following initialization. This refined definition captures trajectories whose strongest coherence reemerges at later times, providing a more faithful correspondence to the underlying TDVP dynamics. Figures S14**b** and **c** compare the two diagnostics side by side for $J_d = 0$: panel **b** shows the first revival peak $\mathcal{I}_{\boldsymbol{z}}^{\max}$, while panel **c** shows the refined maximal revival $\max_{t>t_1} \mathcal{I}_{\boldsymbol{z}}(t)$. The resulting refined Poincaré section, shown in Fig. S14**c**, exhibits markedly improved agreement with the TDVP prediction, correctly recovering the high-stability regions near the top and bottom of the parameter space that were previously underestimated. This demonstrates that the discrepancies stem mainly from focusing on the first revival and ignoring the full periodic structure of the imbalance dynamics, rather than from fundamental deficiencies of the imbalance-based measurement protocol.

To assess the robustness of this conclusion, we repeat the analysis in the presence of a nearest-neighbor diagonal perturbation $J_d = 0.1$, which explicitly breaks the integrability of the TDVP equations. The time series in Fig. S14**d** show that the revival structure persists qualitatively, although the fidelity peaks for $\theta = \pi/4$ are slightly suppressed compared to the unperturbed case. The corresponding Poincaré section maps (Figs. S14**e**,**f**) confirm that the regular islands shrink under the perturbation, consistent with KAM theory, while the chaotic sea expands. Importantly, the refined diagnostic (panel **f**) continues to provide sharper contrast between regular and chaotic regions than the first-revival map (panel **e**), demonstrating that the improved protocol remains effective even when the underlying classical dynamics is partially chaotic.

S11. NUMERICAL METHODS

This section contains an overview of the numerical methods used throughout the paper, whose summary can be found in Table S1. We utilize exact diagonalization to simulate finite-chain dynamics, matrix-product-state TEBD simulations (both finite and infinite) for systematic finite-size scaling, and the hybrid feedback map that combines TEBD with classical nonlinear optimization. Unless specified otherwise, all simulations employ the SSH Hamiltonian of the main text, which in the 1D array geometry can be written as

$$H = \sum_j J_j \left(S_j^+ S_{j+1}^- + \text{h.c.}\right) + J_3 \sum_{j\in\text{odd}} \left(S_j^+ S_{j+3}^- + \text{h.c.}\right), \tag{S37}$$

with alternating nearest-neighbour couplings $J_j = J_o$ on odd (strong dimer) bonds and $J_j = J_e$ on even (weak inter-dimer) bonds. We work at half filling and set $J_o = 1$ as the energy unit, then $J_e = 0.6\, J_o$, $J_3 = 0.05\, J_o$. Time is measured in units of $1/J_o$ and the default Trotter step is $\mathrm{d}t = 0.05$.

A. Exact diagonalization

For finite-size reference dynamics we use exact diagonalization of an $L = 16$ chain, in both periodic and open boundary conditions, at half filling. Under PBCs, we further restrict to the $k = 0$ momentum sector with period-4 sublattice, which is the symmetry sector corresponding to the shallow-circuit ansatz states. This reduces the Hilbert-space dimension from 12870 to 3238. No spatial symmetry is available under OBC and we keep the full half-filling sector.

We diagonalize the Hamiltonian once, and reuse the eigenbasis to build the one-step evolution operator and the state-preparation ramp. The ramp with $t_0 = 1.5/J_o$ is applied once before the evolution in the revival-scan figures to match the experimental state-preparation convention, while we omit it in the co-moving imbalance and time-series figures.

The central observable is the rotated imbalance

$$I_z(\theta,\phi_1,\phi_2;t) = \langle\psi(t)|\, U(\theta,\phi_1,\phi_2)\, M\, U^\dagger(\theta,\phi_1,\phi_2)\, |\psi(t)\rangle\,, \tag{S38}$$

where M is a bare local σ^z-type operator (staggered magnetization under PBC) and $U(\theta,\phi_1,\phi_2)$ is the shallow variational circuit introduced in the main text. Under PBC we absorb the circuit conjugation into a single 16×16 full-site operator acting on each four-site unit cell and average over cells. Under OBC the observable is instead restricted to a four-site window centred on the chain midpoint: we start at site $L/2-1$ and choose the cell type by $L \bmod 8$ (ABCD for $L\equiv 4$, CDAB for $L\equiv 0$) so that the inner pair always lands on the geometric midpoint $(L/2, L/2+1)$.

Typically, we use a 241×161 grid in $(\theta,\phi_2)\in[0,\pi]\times[0,\pi]$ with $\phi_1 = 0$, $\mathrm{d}t = 0.05$, 200 steps ($T_{\max} = 10$), and the ramp $t_0 = 1.5$. The co-moving imbalance of Fig. 2e uses a coarser 61×41 grid with no ramp, evolved to $T = \pi$ before the co-moving observable is evaluated at the TDVP-predicted parameters. The representative time series of Fig. S14 are generated at two seeds, $\theta\in\{0,\pi/4\}$ with $\phi_1=\phi_2=0$, and the same 200-step budget without ramp.

B. Matrix-product-state methods

For the finite-size scaling analysis in Sec. S6 we use TEBD evolution of the shallow-circuit ansatz at chain lengths $L\in\{8,12,16,20,24\}$ with OBC. The ansatz admits an exact MPS representation of bond dimension $\chi_{\text{ansatz}} = 4$ on a four-site unit cell $[A,B,C,D]$; the tensors are constructed in closed form from (θ,ϕ_1,ϕ_2), and the resulting state is orthogonalized and normalized before evolution.

Time evolution uses a second-order Trotter TEBD at $\mathrm{d}t = 0.05/J_o$, with a maximum bond dimension $\chi_{\max} = 64$ and singular-value cutoff 10^{-12}. The rotated imbalance is evaluated on the same centred four-site OBC window used for ED, with a shared helper selecting the ABCD/CDAB cell type so that $L = 8$ uses CDAB, $L = 12$ uses ABCD, and so on.

The thermodynamic-limit reference curves and the entanglement-entropy dynamics are obtained from translation-invariant iMPS simulations. We construct the infinite ansatz starting from a two-site Néel state $|0101\ldots\rangle$, apply a layer of four-site $e^{i\theta XY}$ gates (on sites 1234, then on 2341), and follow with the sublattice-selective $e^{i\phi_k XY}$ rotations. Evolution is then performed with a second-order Trotter splitting of the four-site unit-cell Hamiltonian

$$H_4 = \tfrac{J_o}{2}\big(XY\otimes \mathbb{1}_4 + \mathbb{1}_4\otimes XY\big) + J_e\,\mathbb{1}_2\otimes XY\otimes\mathbb{1}_2 + J_3\big(\sigma_A^+\sigma_D^- + \text{h.c.}\big), \tag{S39}$$

with alternating applications on the two bond sublattices. The default bond dimension is $\chi_{\max} = 64$; for the entanglement-entropy curves of Sec. S9 we converge separately at $\chi_{\max} = 256$.

C. Hybrid feedback map

The Δt-scaling convergence is generated by a hybrid-feedback discrete map on the variational manifold. One step proceeds as follows. The current state is evolved by Δt under the full many-body Hamiltonian using the iTEBD gate of Sec. S11 B. We then measure, on the evolved iMPS, the fourteen local correlators that enter the closed-form expression for the rotated imbalance: four single-site σ^z, six nearest- and next-nearest-neighbour XY, and four three-

Table S1. Numerical settings used in this work. χ denotes MPS bond dimension, d_H the ED Hilbert-space dimension used, $T_{\max}$ the simulated time in units of $1/J_o$.

Method	System	L	Boundary condition (BC)	dt	χ or d_H	$T_{\max}$
ED (Sec. S11 A)	1D SSH	16	PBC	0.05	$d_H = 3238$	
Finite MPS (Sec. S11 B)	1D SSH	8–24	OBC	0.05		
iTEBD (Sec. S11 B)	1D SSH	∞	—	0.05		
Hybrid map (Sec. S11 C)	1D SSH iMPS	∞	—	$\Delta t\in[0.001, 0.1]$	$\chi = 64$	$N \le 1000$ steps

and four-site XZX and $XZZY$ correlators. These determine the imbalance through

$$\begin{aligned} I_z(\theta,\phi_1,\phi_2) = & -\cos(2\theta)\big[\cos(2\phi_1)\,(z_1{-}z_2) + \cos(2\phi_2)\,(z_3{-}z_4)\big] \\ & + 2\cos(2\theta)\big[\sin(2\phi_1)\,\mathrm{xy}_1 + \sin(2\phi_2)\,\mathrm{xy}_2\big] \\ & - 2\sin(2\theta)\cos\phi_1\cos\phi_2\,(\mathrm{xy}_3 + \mathrm{xy}_4) \\ & - 2\sin(2\theta)\sin\phi_1\sin\phi_2\,(\mathrm{xy}_5 + \mathrm{xy}_6) \\ & + 2\sin(2\theta)\sin\phi_1\cos\phi_2\,(\mathrm{xx}_1 - \mathrm{xx}_2) \\ & + 2\sin(2\theta)\sin\phi_2\cos\phi_1\,(\mathrm{xx}_3 - \mathrm{xx}_4). \end{aligned} \tag{S40}$$

We maximize Eq. (S40) over (θ,ϕ_1,ϕ_2) with L-BFGS, warm-started from the previous step's parameters. Each $\phi_1 \to \phi_1 + 2\pi$ crossing is detected by linear interpolation and produces one Poincaré-section point. Every ten steps we re-prepare the iMPS from the fitted parameters to counteract the truncation error that otherwise accumulates along the trajectory. The convergence sweep uses $\Delta t \in \{0.001, 0.005, 0.01, 0.05, 0.1\}$ with a cap of $N = 1000$ iterations per trajectory.

[1] F. Jin, S. Jiang, X. Zhu, Z. Bao, F. Shen, K. Wang, Z. Zhu, S. Xu, Z. Song, J. Chen, Z. Tan, Y. Wu, C. Zhang, Y. Gao, N. Wang, Y. Zou, A. Zhang, T. Li, J. Zhong, Z. Cui, Y. Han, Y. He, H. Wang, J.-N. Yang, Y. Wang, J. Shen, G. Liu, J. Deng, H. Dong, P. Zhang, W. Li, D. Yuan, Z. Lu, Z.-Z. Sun, H. Li, J. Zhang, C. Song, Z. Wang, Q. Guo, F. Machado, J. Kemp, T. Iadecola, N. Y. Yao, H. Wang, and D.-L. Deng, Topological prethermal strong zero modes on superconducting processors, Nature **645**, 626 (2025).

[2] C. M. Langlett, Z.-C. Yang, J. Wildeboer, A. V. Gorshkov, T. Iadecola, and S. Xu, Rainbow scars: From area to volume law, Phys. Rev. B **105**, L060301 (2022).

[3] H. Dong, J.-Y. Desaules, Y. Gao, N. Wang, Z. Guo, J. Chen, Y. Zou, F. Jin, X. Zhu, P. Zhang, H. Li, Z. Wang, Q. Guo, J. Zhang, L. Ying, and Z. Papić, Disorder-tunable entanglement at infinite temperature, Science Advances **9**, eadj3822 (2023).

[4] G. Vidal, Classical simulation of infinite-size quantum lattice systems in one spatial dimension, Phys. Rev. Lett. **98**, 070201 (2007).

[5] E. H. Lieb and D. Robinson, The finite group velocity of quantum spin systems, Commun. Math. Phys. **28**, 251 (1972).

[6] D. S. Bhakuni, R. Verdel, C. Muzzi, R. Andreoni, M. Aidelsburger, and M. Dalmonte, Diagnosing quantum transport from wave function snapshots, Phys. Rev. B **110**, 144204 (2024).

[7] A. Hudomal, A. Daniel, T. S. do Espirito Santo, M. Kornjača, T. Macrì, J. C. Halimeh, G.-X. Su, A. Balaž, and Z. Papić, Ergodicity breaking meets criticality in a gauge-theory quantum simulator (2025), arXiv:2512.23794 [cond-mat.quant-gas].

[8] S. Strogatz, *Nonlinear Dynamics and Chaos: With Applications to Physics, Biology, Chemistry, and Engineering*, Studies in Nonlinearity (Avalon Publishing, 2014).
